# An Explainable Self‐Supervised Temporal Transformer for Participant‐Level Classification of Depressive Symptoms From Resting‐State EEG

**DOI:** 10.1002/mpr.70112

**Published:** 2026-10-13

**Authors:** Pushpamala Ramaiah, Ibtesam Nomani, Hayam Ibrahim Asfour, Mirza R. Baig, Maksudjan Yuldashev, Ibrokhim Sapaev, Zein Bassam Bani Younes, Sumit Sharma, Mahesh Manchanda, Mohamad Ahmad Saleem Khasawneh, Majid S. Jabir

**Affiliations:** ^1^ Faculty of Nursing Umm Al‐Qura University Makkah Saudi Arabia; ^2^ Department of Pharmacy Practice College of Pharmacy Dubai Medical University Dubai UAE; ^3^ Jizzakh State Pedagogical University Education of Management Jizzakh Uzbekistan; ^4^ Head of the Department «Physics and Chemistry» “Tashkent Institute of Irrigation and Agricultural Mechanization Engineers” National Research University Tashkent Uzbekistan; ^5^ Scientific Researcher of the University of Tashkent for Applied Science Tashkent Uzbekistan; ^6^ School of Engineering Central Asian University Tashkent Uzbekistan; ^7^ Western Caspian University Scientific Researcher Baku Azerbaijan; ^8^ Faculty of Arts and Languages / Department of English Language and Literature/Translation Jadara University Irbid Jordan; ^9^ Department of Computer and Science Engineering Chandigarh University Mohali Punjab India; ^10^ Department of Computer Science & Applications Sharda School of Computing Science and Engineering Sharda University Greater Noida India; ^11^ Special Education Department College of Education King Khalid University Abha Saudi Arabia; ^12^ Department of Psychology University of Tehran Tehran Iran

**Keywords:** BDI, deep learning, depressive symptoms, explainable artificial intelligence, resting‐state EEG, self‐supervised learning, SHAP, temporal transformer

## Abstract

**Background:**

Resting‐state electroencephalography (EEG) has emerged as a promising non‐invasive modality for investigating depressive symptoms; however, conventional deep‐learning methods often require large labeled datasets and provide limited interpretability. We developed an Explainable Self‐Supervised Temporal Transformer Network for participant‐level classification of BDI‐defined elevated depressive symptoms from resting‐state EEG.

**Methods:**

The framework employed masked temporal modeling for self‐supervised pretraining followed by supervised fine‐tuning. Model performance was evaluated using repeated nested participant‐wise cross‐validation and compared with conventional machine‐learning and deep‐learning baselines. Explainability was investigated through transformer attention maps and SHAP‐based channel attribution.

**Results:**

The proposed model achieved a receiver operating characteristic area under the curve (ROC‐AUC) of 0.82 (95% CI: 0.73–0.90), a precision–recall AUC of 0.69, balanced accuracy of 0.75, sensitivity of 0.72, and specificity of 0.78. The positive predictive value was 0.57, indicating a substantial false‐positive burden in the evaluated cohort. The proposed model showed the highest point‐estimate ROC‐AUC among the evaluated baselines, with a 0.07 improvement over EEGNet; however, the overlapping uncertainty intervals and the small elevated‐symptom cohort warrant cautious interpretation of comparative superiority. Performance decreased from an ROC‐AUC of 0.82 in Run 1 to 0.78 in Run 2 and to 0.72–0.73 under cross‐condition transfer, supporting interpretation of these analyses as internal reproducibility and condition‐shift assessments rather than independent external validation. Removing self‐supervised pretraining reduced the ROC‐AUC to 0.74. Explainability analyses identified distributed temporally relevant intervals and frontal‐midline to centro‐parietal channel contributions, with attribution interpreted as model‐relevance evidence rather than causal biomarker evidence.

**Conclusion:**

Overall, the findings provide preliminary internal evidence that self‐supervised temporal representation learning can support participant‐level classification of BDI‐defined elevated depressive symptoms from resting‐state EEG under the evaluated experimental conditions. The findings do not establish clinical diagnostic validity, screening readiness, or independent external generalizability.

## 1 Introduction

Major depressive disorder (MDD) is a prevalent and disabling psychiatric condition characterized by persistent affective, cognitive, and somatic symptoms that can substantially impair social and occupational functioning. Depression is also associated with reduced quality of life, increased healthcare utilization, suicide risk, and premature mortality, contributing substantially to the global burden of disease (Santomauro et al. 2021; Ferrari et al. 2022). Despite its clinical importance, the identification and characterization of depressive symptoms remain dependent largely on clinical interviews, behavioral assessment, and self‐report instruments such as the Beck Depression Inventory (BDI). These approaches are essential for clinical assessment but may be influenced by recall bias, symptom underreporting, cultural and contextual factors, clinician‐dependent interpretation, and the marked heterogeneity of depressive phenotypes (Fried and Nesse 2015). Objective neurophysiological measurements could therefore complement conventional assessments by providing quantitative information that may improve the reproducibility and consistency of symptom characterization.

Electroencephalography (EEG) is a particularly attractive modality for this purpose because it is non‐invasive, relatively inexpensive, widely accessible, and provides millisecond‐scale temporal resolution. Resting‐state EEG is especially suitable for participant‐level analysis because it captures spontaneous neural activity without requiring the performance of an explicit cognitive or emotional task. This may be advantageous for individuals experiencing fatigue, psychomotor slowing, impaired concentration, or reduced motivation. Previous studies have reported associations between depressive symptoms and alterations in spectral activity, signal complexity, functional connectivity, and hemispheric asymmetry (de Aguiar Neto and Rosa 2019; Newson and Thiagarajan 2019; Ahmed et al. 2024). Frontal and prefrontal activity has received particular attention because these networks contribute to affective regulation, approach‐withdrawal behavior, reward processing, and cognitive control. However, the direction and magnitude of reported EEG alterations vary across samples, recording conditions, preprocessing procedures, and analytical strategies. This heterogeneity limits the consistency with which individual EEG characteristics can be treated as stand‐alone biomarkers and motivates data‐driven approaches that can account for complex and temporally varying neural patterns.

Conventional computational approaches to EEG‐based depression *analysis* commonly rely on manually engineered features, including spectral power, hemispheric asymmetry, entropy, fractal measures, and functional‐connectivity indices, followed by statistical or machine‐learning classifiers such as support vector machines, random forests, and shallow neural networks. Although these approaches have generated encouraging findings, their performance depends substantially on feature‐selection and feature‐engineering decisions, as well as on prior assumptions concerning which EEG characteristics are informative (de Aguiar Neto and Rosa 2019). Handcrafted representations may also compress highly dynamic neural recordings into relatively static summaries, potentially discarding transient temporal structure and interactions occurring across multiple time scales. Such limitations are particularly relevant to resting‐state EEG, where neural activity continuously evolves and clinically relevant patterns may be distributed across both local and long‐range temporal dependencies.

Deep learning provides an alternative by learning hierarchical representations directly from raw or minimally processed EEG recordings. Convolutional neural networks (CNNs) can capture local temporal and spatial patterns, whereas recurrent architectures can represent sequential dependencies across samples or temporal windows. Reviews of EEG deep‐learning research indicate that learned representations can provide advantages over conventional feature‐based pipelines when sufficient data and rigorous participant‐level evaluation are available (Craik et al. 2019; Roy et al. 2019). However, most deep‐learning systems for EEG remain predominantly supervised. Reliable clinical labels are often expensive to obtain because EEG acquisition requires specialized equipment and expertise, while symptom assessment and annotation may require substantial clinical effort. In relatively small datasets, high‐capacity supervised models may consequently learn subject‐specific characteristics, recording artifacts, or other dataset‐dependent signals rather than representations that remain stable across participants and recording conditions. This issue is particularly important in psychiatric neuroimaging, where sample sizes are often considerably smaller than those typically required to train high‐capacity models from scratch.

Self‐supervised learning provides a potentially useful strategy for improving data efficiency by exploiting the internal structure of unlabeled physiological signals to construct training objectives without requiring diagnostic labels. In a typical self‐supervised framework, a model is first pretrained using tasks such as masked‐signal reconstruction, temporal‐context prediction, or contrastive learning and is subsequently fine‐tuned on a smaller labeled dataset for the downstream task. Previous EEG studies have shown that self‐supervised representation learning can improve data efficiency and provide useful representations for subsequent subject‐level prediction tasks (Kostas et al. 2021; Banville et al. 2021). Masked temporal modeling is particularly compatible with resting‐state EEG because the model is required to infer missing portions of a continuous signal from surrounding temporal context. This encourages learning relationships among neighboring and more distant signal segments without constructing artificial clinical labels.

More broadly, annotation‐efficient and domain‐robust learning strategies have increasingly been investigated in biomedical artificial‐intelligence applications because models trained under one data distribution may encounter substantial variation in acquisition protocols, populations, and imaging or physiological conditions. For example, unsupervised domain‐adaptation approaches have been used to reduce reliance on extensive annotations while improving transfer across heterogeneous biomedical data distributions (He et al. 2023). Although such work is not specific to EEG or depression, it highlights a broader methodological principle that is relevant to physiological signal analysis: representation learning should be evaluated not only by performance under a single experimental condition but also by its sensitivity to changes in data distribution. In the present study, this consideration is particularly relevant because resting‐state EEG recordings can differ between eyes‐open and eyes‐closed conditions, which may alter spectral composition and temporal dynamics.

Transformers provide a suitable architecture for self‐supervised temporal modeling because their self‐attention mechanism can capture dependencies between distant elements of a sequence (Vaswani et al. 2017). Unlike conventional recurrent networks, which process observations sequentially and may have difficulty retaining information across long intervals, transformers model relationships among all temporal tokens directly. Recent transformer‐based EEG architectures have demonstrated the potential of attention mechanisms for jointly learning local signal characteristics and long‐range dependencies (Song et al. 2022). However, their application to participant‐level depressive‐symptom classification remains comparatively limited. Many studies continue to use short independent epochs, supervised training, or static time‐frequency representations, thereby underutilizing the continuous temporal organization of resting‐state brain activity. In addition, high classification performance reported at the EEG‐segment level may not translate to participant‐level performance when segments from the same participant are inadvertently distributed across training and testing sets. Such leakage can result in overly optimistic estimates because the model may exploit participant‐specific characteristics that persist across windows. Therefore, participant‐wise separation between training and evaluation data is essential when assessing the performance of EEG‐based models intended for participant‐level inference.

Interpretability represents a further barrier to the clinical adoption of deep neural networks. A model that produces a diagnostic probability without indicating the neural evidence underlying its decision offers limited clinical and scientific value. Attention visualization can indicate the temporal windows to which a transformer assigns greater importance, but attention weights alone should not automatically be interpreted as complete explanations of model behavior (Jain and Wallace 2019). Complementary post‐hoc attribution methods can therefore provide additional information. SHapley Additive exPlanations (SHAP) provide a theoretically grounded approach for estimating the contribution of individual input features to a prediction (Lundberg and Lee 2017). In multichannel EEG analysis, SHAP‐based attribution can quantify the relative contribution of electrodes or brain regions, whereas attention analysis can reveal temporally salient signal intervals. Combining these techniques may provide complementary temporal and spatial perspectives on model decisions and can facilitate model auditing and hypothesis generation.

Neurophysiological plausibility should nevertheless be distinguished from causal or clinical validity. Frontal EEG asymmetry has long been investigated as a possible marker of vulnerability to depression, although its reliability is influenced by methodological and individual factors (Allen and Reznik 2015). A meta‐analysis similarly reported relationships between depression, anxiety, and resting frontal EEG asymmetry while emphasizing heterogeneity across studies (Thibodeau et al. 2006; Monni et al. 2022). Consequently, the identification of frontal channel importance by an explainable model may support consistency with prior evidence, but it should not be interpreted as proof of a causal biomarker. In the present framework, attribution results are therefore treated as model‐derived descriptive evidence that can support interpretation, model auditing, and generation of hypotheses for future experimental investigation.

Despite progress in EEG‐based deep learning, an integrated framework that combines label‐efficient representation learning, temporal modeling, participant‐level evaluation, and complementary model interpretation remains relatively underexplored for depressive‐symptom classification. Existing approaches commonly address only one or two of these requirements: supervised CNN and recurrent models depend heavily on labeled data; transformer models may not exploit self‐supervised pretraining; and explainability is frequently limited to a single post‐hoc method. Moreover, apparent performance stability within a single recording condition does not necessarily imply robustness to changes in recording condition or independent generalizability to new participants and acquisition settings. In particular, eyes‐open and eyes‐closed resting‐state recordings may introduce distributional differences in EEG dynamics that provide an informative test of within‐dataset reproducibility. These considerations motivate evaluation procedures that explicitly distinguish participant‐wise reproducibility from external generalizability.

To address these limitations, this study proposes an Explainable Self‐Supervised Temporal Transformer Network for participant‐level classification of BDI‐defined elevated depressive symptoms using resting‐state EEG brain dynamics. The proposed framework leverages self‐supervised masked temporal modeling to learn informative EEG representations without diagnostic labels, followed by supervised fine‐tuning for binary classification. To improve model interpretability, attention‐weight visualization and SHAP‐based channel attribution are incorporated to provide complementary temporal and spatial explanations of the model's predictions. The framework is evaluated using a publicly available resting‐state EEG cohort collected from young adults with a broad range of depressive symptom severity under a rigorous subject‐wise cross‐validation protocol. In addition, performance is examined across resting‐state recording conditions to assess internal reproducibility under changes in acquisition condition. These analyses are intended to characterize the behavior of the model within the available cohort rather than to establish independent external generalizability or clinical diagnostic validity.

The main contributions of this study are as follows:

1. A label‐efficient self‐supervised representation learning strategy that uses masked temporal modeling to learn representations from resting‐state EEG before supervised participant‐level classification, reducing reliance on symptom‐labeled data during representation learning.
2. A temporal transformer architecture designed to capture both short‐term fluctuations and longer‐range temporal dependencies in spontaneous brain dynamics.
3. A strict subject‐wise evaluation protocol that prevents segments from the same participant from appearing in both training and testing folds, thereby reducing the risk of participant‐level information leakage.
4. An explainability framework combining attention visualization with SHAP‐based channel attribution to provide complementary temporal and spatial descriptions of model behavior. These attribution results are interpreted as model‐behavior evidence rather than as causal biomarkers.
5. A comparative evaluation against CNN and CNN–LSTM baselines, including analysis across eyes‐open and eyes‐closed resting‐state conditions to characterize internal reproducibility and sensitivity to recording‐condition variation.

## 2 Materials and Methods

### 2.1 Study Design

This study was designed as a retrospective, participant‐level EEG classification study using a publicly available resting‐state EEG cohort. The primary objective was to determine whether self‐supervised temporal representation learning could discriminate participants with BDI‐defined elevated depressive symptoms from participants with low depressive symptom scores. The study was conducted as a secondary analysis of an openly available dataset and was reported according to the principles of the TRIPOD + AI framework.

The participant, rather than the individual EEG segment, was the fundamental unit of analysis. EEG windows were treated as repeated observations nested within participants and were therefore never distributed across different training, validation, or test partitions. This design was used to prevent participant‐specific signal characteristics from producing artificially optimistic estimates of model performance.

The primary analysis used the first resting‐state EEG session (Run 1) and evaluated participant‐level discrimination under a strictly subject‐wise cross‐validation framework. Three predefined secondary analyses were conducted. First, temporal reproducibility was examined by applying the model trained using Run 1 to the independently acquired second resting‐state session (Run 2) without model retraining, recalibration, or threshold adjustment. Second, model predictions were compared with structured clinical interview status in the subset for whom interview information was available. Third, performance was evaluated separately for eyes‐open and eyes‐closed resting conditions, including cross‐condition transfer analyses.

These secondary analyses were interpreted as internal reproducibility, clinical‐reference comparison, and within‐dataset condition‐shift analyses, respectively. None was considered evidence of independent external validation because all analyses were derived from the same study cohort, recording site, and acquisition environment.

Before model fitting, the analysis framework specified participant eligibility, outcome construction, EEG quality‐control criteria, preprocessing procedures, segmentation, normalization, model architecture, hyperparameter‐search ranges, probability aggregation, threshold selection, calibration, evaluation metrics, statistical comparison procedures, and prespecified sensitivity analyses. All file‐level exclusions, quality‐control decisions, and annotation corrections were recorded in machine‐readable processing logs to ensure reproducibility.

### 2.2 Data Source

Data were obtained from the openly available OpenNeuro dataset ds003478, version 1.1.0 (DOI: 10.18112/openneuro.ds003478.v1.1.0) (Cavanagh 2021). The dataset contains resting‐state EEG recordings from 122 college‐age participants and is distributed under a CC0 license in BIDS format. Independent dataset documentation confirms 243 EEG recordings, a nominal sampling rate of 500 Hz, and two resting‐state sessions per participant, with the second session recorded after an intervening task period.

The recordings were collected at the University of Arizona between approximately 2008 and 2010. Participants underwent resting‐state EEG acquisition consisting of alternating approximately one‐minute eyes‐open and eyes‐closed intervals. The first resting‐state session was acquired immediately after EEG setup, whereas the second session was acquired after approximately 1 hour of intervening task performance. Participant sub‐516 had no second resting‐state recording. Dataset documentation also identifies potential HEOG/VEOG label reversals, the presence of channels that had already been interpolated before public release, and limited quality characterization of the second rest session.

Because the public repository does not provide access to uninterrupted pre‐interpolation recordings, previously interpolated channels could not be reconstructed from raw data. Accordingly, these channels were retained as documented by the source dataset and were explicitly flagged for sensitivity analysis rather than silently altered.

The dataset documentation further indicates that sub‐544 was excluded because of unstable BDI classification between assessment time points, whereas sub‐516 lacked the second resting‐state session. These provenance characteristics were incorporated directly into the participant‐flow logic and secondary‐analysis definitions. The dataset identifier, version, DOI, download date, and repository commit or snapshot identifier were retained as part of the reproducibility record.

### 2.3 Outcome Definition and Participant Eligibility

Depressive symptom severity was determined from BDI measurements provided in the participant‐level clinical metadata and associated assessment files. To avoid converting a dimensional symptom measure into an unsupported psychiatric diagnosis, the primary outcome was explicitly defined as elevated depressive symptoms rather than major depressive disorder.

A binary outcome was prespecified using the available BDI score distribution. Participants with BDI scores ≤ 13 were classified as the low‐symptom group, whereas participants with BDI scores ≥ 20 were classified as the elevated‐symptom group. Participants with scores from 14 to 19 were considered intermediate and were excluded from the primary binary analysis because these values represented an intermediate symptom range that was less suitable for an extreme‐group binary contrast.

Before EEG quality control, the available clinical data comprised 76 participants with low symptom scores, 30 participants with elevated symptom scores, and 16 participants with intermediate scores. One participant with documented instability in BDI classification between assessment time points was excluded before EEG modeling. The 16 intermediate‐BDI participants were retained for prespecified sensitivity analyses. Following EEG quality control, three additional low‐symptom participants were excluded, resulting in a final primary analytical cohort of 73 low‐symptom participants and 29 elevated‐symptom participants (*n* = 102).

This participant‐flow structure was maintained consistently throughout the analysis: the 16 intermediate‐BDI participants were excluded from the primary binary analysis but retained for sensitivity analyses; one participant with unstable BDI classification was excluded before EEG modeling; and three low‐symptom participants were subsequently excluded following EEG quality control.

BDI values were cross‐checked across the available assessment fields. When conflicting values were identified, the prespecified source‐of‐truth field used in the original dataset documentation was retained. Missing clinical values were not imputed. After outcome construction, subsequent exclusions were based only on predefined EEG quality or data‐availability criteria.

Because BDI grouping does not establish the presence or absence of a formal psychiatric disorder, all primary analyses refer to BDI‐defined depressive‐symptom groups. In the subset with structured interview information, model predictions were separately compared with interview‐derived clinical status. The interview analysis was treated as a clinical‐reference comparison, not as definitive diagnostic validation of the EEG model.

Demographic and recording characteristics were summarized for each outcome group, including age, sex, BDI score, interview availability, Run 1 and Run 2 availability, usable recording duration, retained EEG channel count, and the proportion of rejected EEG windows. Continuous variables were summarized using mean ± standard deviation or median and interquartile range, as appropriate, and compared using Welch's *t*‐test or the Mann–Whitney *U* test. Categorical variables were summarized as counts and percentages and compared using Fisher's exact test.

To assess whether model predictions could be explained by non‐neural characteristics, secondary analyses examined associations between participant‐level predicted probability and age, sex, usable recording duration, retained‐channel count, and window‐rejection proportion. These analyses were treated as potential‐confounding assessments rather than as primary predictive outcomes.

### 2.4 EEG Audit and Condition Annotation

Before preprocessing, a deterministic validation pipeline inspected every EEG recording and its associated BIDS metadata, channel file, event file, and sidecar information. The audit verified file readability, sampling frequency, recording duration, channel count and naming, event ordering and duration, duplicated samples, non‐finite values, and consistency between EEG recordings and metadata.

BIDS validation warnings were retained together with their severity and affected files. Warnings judged to be non‐fatal were documented but did not automatically result in exclusion, whereas conditions that violated prespecified signal‐integrity requirements resulted in deterministic exclusion.

Eyes‐open and eyes‐closed intervals were reconstructed from the event annotations using a predefined condition‐mapping table. Each usable interval was required to have a valid onset, a positive duration, and no overlap with another condition interval. An EEG window was assigned a condition label only when the entire two‐second window was contained within a valid condition interval. Windows crossing instruction boundaries, condition transitions, or incompletely annotated intervals were excluded from condition‐specific analyses.

Recordings with incomplete condition annotation, including the documented annotation issue affecting sub‐033 Run 1, remained eligible for condition‐agnostic analyses when the underlying EEG passed signal‐quality criteria. They were excluded from condition‐specific analyses when the condition sequence could not be established unambiguously from the available event information. Reconstructed labels were flagged separately and were evaluated only in the corresponding sensitivity analysis.

Potential HEOG/VEOG mislabeling was handled without relying exclusively on channel names. Channel metadata were cross‐referenced with temporal and spatial signal characteristics. Blink‐related activity, frontal‐channel correlation, and low‐frequency ocular signatures were used to identify the likely vertical EOG component, whereas lateralized ocular activity and opposing horizontal topographic patterns were used to identify the likely horizontal EOG component. Original channel labels and algorithmically inferred EOG roles were both retained in the quality‐control record.

### 2.5 EEG Preprocessing and Quality Control

All preprocessing was performed independently for each recording using MNE‐Python. Continuous EEG was converted to double precision and band‐pass filtered from 1 to 40 Hz using a zero‐phase finite‐impulse‐response filter. The primary pipeline did not use a notch filter because the upper passband excluded the nominal 50‐Hz line‐frequency component. A 50‐Hz notch‐filter sensitivity analysis was prespecified for recordings showing residual line‐noise contamination after primary preprocessing.

Channels identified by the source dataset as previously interpolated were retained but flagged. A sensitivity analysis excluded recordings in which more than 10% of scalp EEG channels had already been interpolated before release. Additional bad channels were identified using complementary criteria including prolonged flat activity, abnormal amplitude distribution, low correlation with neighboring channels, excessive high‐frequency noise, and outlying log‐variance. A channel was considered unsuitable when at least two independent quality criteria were simultaneously satisfied. When valid electrode coordinates were available, newly identified bad channels were interpolated using spherical‐spline interpolation.

Recordings were excluded from the corresponding analysis when more than 15% of scalp EEG channels were unusable after quality control. Three recordings in the primary cohort met this predefined quality‐control exclusion criterion. The remaining quality‐control procedures included assessment of usable recording duration and excessive channel‐level artifacts according to the predefined recording‐quality criteria. These criteria were applied before model input generation and independently of participant outcome.

Artifact attenuation was performed using independent component analysis (ICA) fitted to a 1‐Hz high‐pass‐filtered copy of each recording. The number of retained ICA components was constrained by the effective signal rank. Candidate ocular, cardiac, and muscle‐related components were identified using a combination of temporal waveform properties, frequency‐domain characteristics, and scalp topography. A component was removed only when at least two predefined indicators supported the same artifact interpretation. All removed components were recorded in the quality‐control log.

After artifact correction, EEG was re‐referenced to the common average. The final cleaned recordings were segmented into analysis windows before residual‐artifact screening.

Residual artifact rejection was performed at the window level. Windows containing non‐finite samples, prolonged flat activity, extreme peak‐to‐peak amplitude, abrupt sample‐to‐sample discontinuities, or marked deviation from the recording‐specific robust variance distribution were rejected. Fixed quality thresholds were combined with within‐recording robust thresholds to accommodate differences in archived signal scaling. The proportion of rejected windows and the number of retained windows were documented for every participant and summarized by outcome group.

Importantly, quality‐control decisions were made independently of the depressive‐symptom label and before model optimization. This was intended to minimize differential preprocessing or outcome‐dependent exclusion.

### 2.6 Data Partitioning, Segmentation, and Normalization

The primary evaluation used repeated nested stratified group cross‐validation. The outer loop consisted of five subject‐disjoint folds repeated across five prespecified random seeds, yielding 25 outer evaluation replicates. Each participant belonged to one outer test fold within a given repetition, and all EEG windows, conditions, and available rest sessions from that participant remained in the same partition.

Outcome stratification was performed at the participant level to preserve the approximate low‐symptom/elevated‐symptom distribution across folds. No window‐level stratification was used.

Within each outer training‐development set, four‐fold subject‐wise inner cross‐validation was used for hyperparameter optimization, early stopping, probability calibration, and threshold determination. The outer test participants were never used for any stage of model selection.

All data‐dependent transformations were fitted exclusively within the corresponding training partition. These included normalization parameters, class weights, feature statistics, calibration parameters, threshold selection, and background samples used for explainability. The resulting transformations were then applied unchanged to the associated validation or outer test participants.

Continuous EEG was segmented into 2‐s windows with a 1‐s stride, corresponding to 1000 samples per window at 500 Hz. Segmentation was performed independently within recording sessions and valid resting‐state conditions. Windows were never allowed to cross condition boundaries.

To prevent participants with longer usable recordings from contributing disproportionately to training, optimization used participant‐balanced sampling. At each training iteration, the contribution of an individual participant was normalized so that the effective sampling probability did not scale linearly with the number of retained windows.

Channel‐wise signal normalization used the median and interquartile range estimated exclusively from the training participants in the corresponding outer‐fold development set. The fitted transformation was applied unchanged to validation and test data. No normalization statistic was calculated using the complete cohort.

### 2.7 Self‐Supervised Temporal Transformer

Each two‐second EEG window was divided into non‐overlapping 50‐ms temporal patches. At the nominal sampling rate of 500 Hz, each patch contained 25 samples, resulting in 40 temporal tokens per EEG window.

Each multichannel temporal patch was linearly projected into a 128‐dimensional embedding space. A learnable classification token was appended to the sequence, and learnable positional embeddings were added to preserve temporal order.

The encoder consisted of four pre‐normalized Transformer blocks, with eight self‐attention heads, an embedding dimension of 128, and a feed‐forward dimension of 512. GELU nonlinearities, residual connections, and a dropout probability of 0.10 were used. These structural dimensions were fixed before outer‐loop evaluation and were not altered using information from the outer test folds.

The parameters subject to inner‐loop optimization included learning rate, masking ratio, weight decay, classification‐head dropout, and the coefficient controlling the spectral component of the pretraining objective. The search was conducted only within participant‐disjoint inner folds.

Self‐supervised pretraining used EEG recordings from training participants only. During pretraining, 40% of the temporal tokens were masked using contiguous mask spans with a mean length of three tokens. The network was trained to reconstruct the masked signal using information from the observed temporal context.

The reconstruction objective combined a time‐domain L1 loss with a spectral‐domain L1 loss computed from the logarithmically transformed spectral representation. The loss was evaluated exclusively over the masked positions. No clinical label was used during this pretraining stage.

After pretraining, the reconstruction head was discarded and the Transformer encoder was fine‐tuned for participant‐level symptom‐group classification using a binary classification head attached to the classification‐token representation.

The supervised objective used participant‐balanced weighted binary cross‐entropy. Class weights were derived from the number of participants rather than the number of EEG windows.

Optimization used AdamW with gradient clipping, linear warm‐up, cosine learning‐rate decay, and early stopping based on inner‐validation participant‐level ROC‐AUC. The outer test set remained inaccessible during all optimization decisions.

### 2.8 Subject‐Level Prediction, Aggregation, Thresholding, and Calibration

The trained model generated a probability estimate for each retained EEG window. To obtain a participant‐level prediction without allowing subjects with longer recordings to dominate the analysis, predictions were aggregated hierarchically.

First, window probabilities were averaged within each valid resting‐state condition for a participant. When both eyes‐open and eyes‐closed predictions were available, the two condition‐level probabilities were assigned equal weight and averaged to obtain the primary participant‐level probability. This procedure ensured that the number of retained windows within a condition did not directly determine the participant's final prediction.

Participants with incomplete condition availability were retained for analyses in which the corresponding condition was not required, with the missing condition documented explicitly. The primary model evaluation used the prespecified aggregation procedure.

The classification threshold was selected within the inner‐development data by maximizing balanced accuracy and was then fixed before application to the corresponding outer test fold. Consequently, sensitivity, specificity, PPV, NPV, F1‐score, and confusion matrices were calculated using fold‐specific thresholds selected without access to the outer test participants.

Confusion matrices were therefore not generated using a single threshold retrospectively optimized from pooled outer‐test predictions.

Probability calibration was performed using temperature scaling fitted only to inner‐validation predictions. Both calibrated and uncalibrated predictions were retained for evaluation, but the primary discrimination analysis was based on ROC‐AUC.

Calibration was evaluated using the Brier score, calibration intercept, calibration slope, and expected calibration error, together with a participant‐level calibration plot. *Five participant‐level probability bins were used for the calibration analysis, and 95% confidence intervals for observed event rates were estimated using participant‐level bootstrap resampling*.

### 2.9 Internal Reproducibility, Clinical‐Reference Comparison, Condition Analysis, and Explainability

The primary outcome was participant‐level ROC‐AUC. Secondary outcomes included precision–recall AUC, balanced accuracy, sensitivity, specificity, F1‐score, positive predictive value, negative predictive value, and Matthews correlation coefficient. Segment‐level metrics were considered supplementary and were not used as evidence of participant‐level clinical performance.

#### 2.9.1 Temporal Reproducibility

To evaluate reproducibility over time, the complete model selected using Run 1 was applied to Run 2 without additional model training, fine‐tuning, recalibration, threshold optimization, or participant‐specific adaptation. The Run‐2 results were therefore designed to assess temporal stability within the same cohort and were interpreted as internal reproducibility rather than independent external validation.

#### 2.9.2 Clinical‐Reference Comparison

For the subset with structured interview information, participant‐level predictions were compared with interview‐derived clinical status. The analysis included ROC‐AUC, the class distribution of the interview‐defined groups, a participant‐level confusion matrix, sensitivity, specificity, positive predictive value, negative predictive value, and corresponding confidence intervals, together with Cohen's kappa.

The interview subset contained 61 participants with available structured interview information. Because agreement between BDI‐defined grouping and interview‐derived status was not complete, the analysis was interpreted as a clinical‐reference comparison rather than definitive diagnostic validation.

#### 2.9.3 Resting‐State Condition Analysis

Condition‐specific evaluation was performed separately for eyes‐open and eyes‐closed resting EEG. In addition, cross‐condition transfer was assessed in both directions: models trained using one resting‐state condition were evaluated using the other condition without fine‐tuning or condition‐specific recalibration.

These analyses were explicitly interpreted as tests of sensitivity to within‐dataset recording‐condition variation. They were not considered external validation because the recordings originated from the same participant population, study site, and acquisition system.

#### 2.9.4 Explainability and Attribution Fidelity

Temporal relevance was examined using attention rollout with residual connections accounted for before combining attention matrices across Transformer blocks. Attention‐derived temporal relevance was summarized at the participant level after hierarchical aggregation across windows.

Channel‐level attribution was estimated using GradientSHAP. The SHAP background distribution consisted exclusively of training‐participant windows from the corresponding fold, thereby preventing information from outer test participants from entering the explanation procedure.

Attributions were aggregated hierarchically across time, windows, and participants. The stability of attribution rankings was examined across outer folds, random seeds, resting‐state sessions, and recording conditions.

To assess attribution fidelity, EEG channels or temporal patches were removed in descending order of absolute attribution magnitude, and the change in model confidence was compared with the change produced by randomly selected deletions matched for the number of removed features.

These analyses were interpreted strictly as model‐relevance and model‐auditing analyses. They were not interpreted as evidence of causal neurophysiological mechanisms or as validation of depression biomarkers.

### 2.10 Statistical Analysis

All primary uncertainty estimates were calculated at the participant level. EEG windows were never treated as independent statistical observations for confidence intervals or hypothesis testing.

For each outer‐fold prediction, participant‐level probabilities were retained before calculation of summary performance statistics. ROC‐AUC, PR‐AUC, sensitivity, and specificity were retained separately for each outer fold and random seed and summarized as fold‐/seed‐level performance distributions.

Ninety‐five percent confidence intervals were calculated using 2000 participant‐level bootstrap resamples, with resampling performed at the participant level rather than at the window level. Bootstrap procedures preserved the paired structure required for within‐participant comparison of competing models.

For direct comparison between the proposed model and each baseline, the primary effect estimate was the paired participant‐level difference in ROC‐AUC, accompanied by its 95% confidence interval. Where appropriate, analogous paired differences were calculated for PR‐AUC and balanced accuracy.

Threshold‐dependent paired comparisons were conducted using participant‐level permutation procedures or McNemar's test when applicable. Confidence intervals for sensitivity, specificity, PPV, and NPV were calculated using bootstrap resampling.

Multiple pairwise comparisons against the baseline models were corrected using the Holm procedure. Two‐sided adjusted *p*‐values below 0.05 were considered statistically significant. Effect magnitude and uncertainty intervals were prioritized over isolated significance values, particularly because the elevated‐symptom group contained only 29 participants.

The proposed model was compared with a supervised Transformer matched for core architecture, a one‐dimensional CNN, a CNN–LSTM, EEGNet, Deep4Net, support vector machine, and random forest. All models were evaluated using the same participant‐level outer folds, the same preprocessed EEG recordings, the same participant‐level aggregation strategy, and the same outcome labels.

Each baseline received its own nested inner‐loop hyperparameter optimization. Hyperparameter searches were restricted to predefined ranges and used only inner‐fold data. Thus, no baseline was granted access to the outer test participants during model selection.

### 2.11 Prespecified Sensitivity Analyses

Several sensitivity analyses were specified to determine whether the primary findings depended on particular preprocessing, segmentation, or cohort‐definition choices.

First, artifact rejection thresholds were made more stringent to determine whether model performance was driven by residual contaminated windows. Second, recordings containing a substantial proportion of channels that had already been interpolated before public release were removed. Third, recordings with uncertain or reconstructed condition annotations were excluded from condition‐specific analyses.

Fourth, the segmentation strategy was varied by comparing the primary 2‐s/1‐s‐stride windows with 1‐s windows, 4‐s windows, and a non‐overlapping segmentation scheme. Fifth, demographic adjustment analyses examined whether adding age and sex information materially altered participant‐level predictions.

Sixth, the intermediate BDI group was incorporated in a predefined sensitivity framework. Two complementary analyses were performed:

1. ordinal symptom‐severity modeling across low, intermediate, and elevated groups; and
2. continuous prediction of BDI score across the full available symptom range.

The intermediate‐group analyses were treated as sensitivity analyses and were not used to replace the primary extreme‐group binary classification. Their purpose was to determine whether the primary AUC could have been influenced by excluding participants with intermediate symptom severity.

Finally, sensitivity analyses compared calibrated and uncalibrated probabilities and evaluated whether the principal conclusions were materially altered when fold‐specific thresholds were replaced by a threshold fixed before evaluation.

### 2.12 Reproducibility, Data Leakage Prevention, and Software

The complete analysis pipeline was implemented in Python using MNE‐Python, PyTorch, scikit‐learn, SciPy, and SHAP. Code was version‐controlled and all analysis stages were executed through reproducible configuration files.

The reproducibility record included the OpenNeuro dataset identifier and version, dataset snapshot or commit information, software versions, preprocessing configuration, quality‐control logs, participant‐level cross‐validation assignments, model hyperparameters, model checkpoints, out‐of‐fold participant predictions, threshold values, calibration parameters, attribution outputs, and statistical‐analysis scripts.

To minimize data leakage, all operations capable of learning from the data—including normalization, hyperparameter selection, early stopping, class weighting, threshold selection, temperature scaling, SHAP background selection, and any data‐dependent model configuration—were restricted to the corresponding training or inner‐validation partitions. No information from outer test participants was used during model development.

### 2.13 Ethics and Scope of Inference

The study used publicly available, de‐identified data collected under the ethical and informed‐consent procedures of the original investigation. No new participant contact, intervention, or collection of identifiable information occurred in the present secondary analysis.

The analytical outcome was classification of BDI‐defined elevated depressive symptoms, not diagnosis of major depressive disorder. Accordingly, the model was developed as a research‐oriented participant‐level stratification framework and was not presented as a standalone diagnostic instrument or clinically validated screening tool.

The temporal reproducibility, clinical‐reference, ablation, and condition‐shift analyses were used to characterize the internal behavior and stability of the model within the available dataset. They do not establish external generalizability. Independent evaluation in a larger cohort containing participants across the full spectrum of depressive symptom severity, preferably with structured diagnostic assessment and independent acquisition sites and equipment, is required before conclusions regarding clinical utility can be drawn.

## 3 Results

### 3.1 Participant Flow and Analytical Cohort

The OpenNeuro ds003478 dataset initially included 122 college‐age participants with resting‐state EEG recordings. Based on the prespecified BDI thresholds, 76 participants were initially classified as low‐symptom controls and 30 participants were classified as having elevated depressive symptoms. Sixteen participants with intermediate BDI scores were excluded from the primary binary classification analysis. One participant with documented instability in BDI classification across the mass‐screening and laboratory assessments was excluded before EEG modeling. Following file‐level validation, event verification, and EEG quality control, three additional low‐symptom participants were excluded according to the predefined EEG quality‐control criteria, resulting in a final analytical cohort of 73 low‐symptom controls and 29 participants with elevated depressive symptoms (*n* = 102).

The final primary analytical cohort included 102 participants, comprising 73 low‐symptom controls and 29 participants with elevated depressive symptoms. Valid second resting‐state recordings were available for 96 participants. Structured interview information was available for 61 participants, including 21 participants with an interview‐confirmed depressive disorder and 40 participants without a current depressive disorder. Valid eyes‐open and eyes‐closed intervals were identified for 99 and 98 participants, respectively. The Run‐1 recording of participant sub‐033 was excluded from condition‐specific analyses because of incomplete eyes‐closed event annotation but was retained in the condition‐agnostic primary analysis after passing EEG quality control.

Figure 1 shows the participant‐selection process from the full OpenNeuro dataset to the final primary and secondary analytical cohorts. The primary cohort contained 102 participants after exclusion of the 16 participants with intermediate BDI scores, the participant with unstable BDI classification, and three participants excluded following EEG quality control. The temporal reproducibility, interview‐reference, and condition‐specific analyses were performed in partially overlapping subsets of the primary cohort.

**FIGURE 1:**
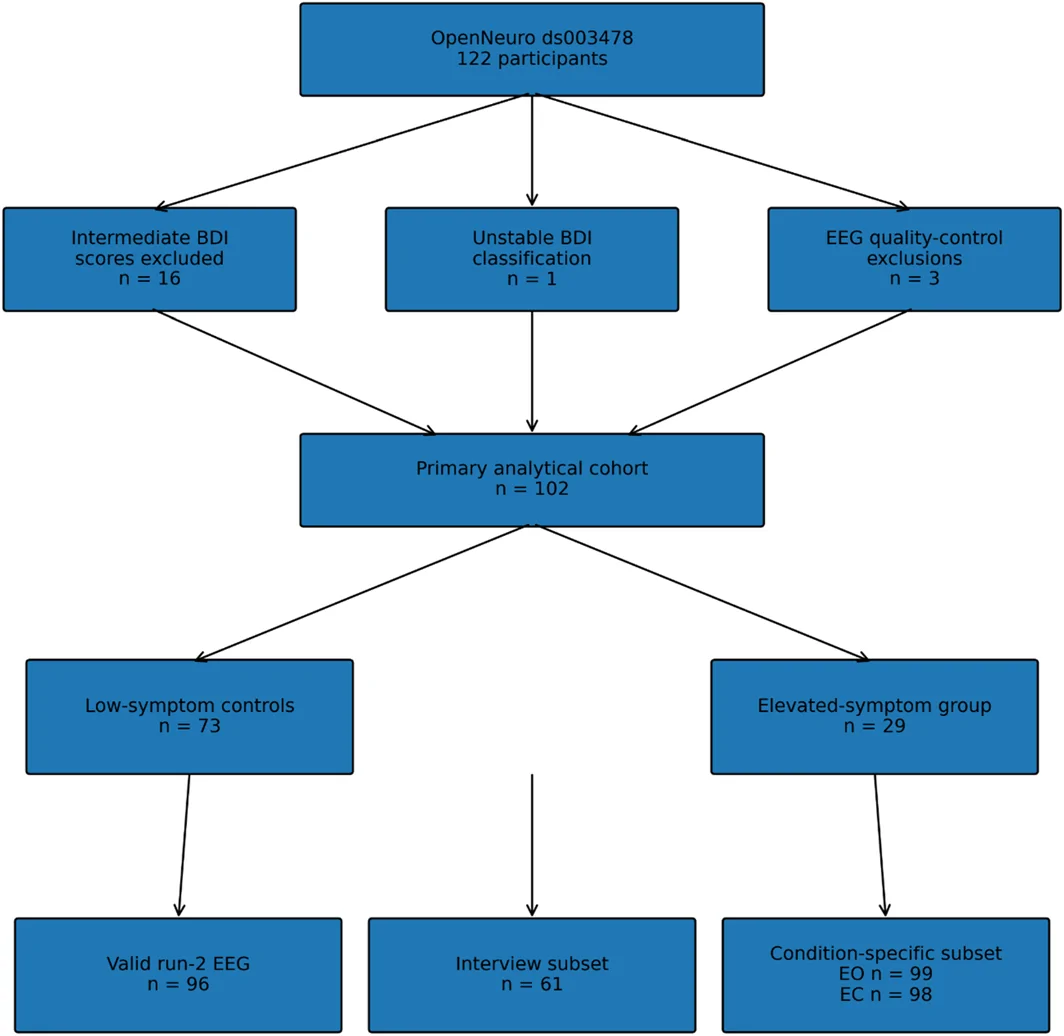
Participant flow and derivation of the analytical cohorts.

Table 1 shows the demographic, clinical, and EEG quality characteristics of the final analytical cohort. The low‐symptom control and elevated‐symptom groups were well balanced with respect to age, sex distribution, interview availability, availability of the second resting‐state EEG recording, channel interpolation, proportion of rejected EEG windows, retained recording duration, and the number of analyzable EEG windows (all *p* > 0.05). As expected, participants in the elevated‐symptom group had substantially higher BDI scores than the low‐symptom controls (*p* < 0.001). The comparable EEG quality metrics between groups indicate that differences in model performance were unlikely to be attributable to systematic variations in recording quality, artifact burden, or the amount of usable EEG data.

**TABLE 1:** Demographic, clinical, and EEG quality characteristics of the final analytical cohort.

Characteristic	Low‐symptom controls (*n* = 73)	Elevated‐symptom group (*n* = 29)	Standardized difference/*p*‐value
Age, years, mean ± SD	20.8 ± 2.1	21.3 ± 2.4	0.22/0.31
Female sex, *n* (%)	45 (61.6%)	19 (65.5%)	0.08/0.72
BDI score, median (IQR)	5 (3–8)	26 (23–31)	< 0.001
Interview data available, *n* (%)	40 (54.8%)	21 (72.4%)	0.37/0.11
Valid Run‐2 EEG recording, *n* (%)	69 (94.5%)	27 (93.1%)	0.06/0.79
Previously interpolated channels, median (IQR)	1 (0–2)	1 (0–2)	0.04/0.85
Newly interpolated channels, median (IQR)	1 (0–2)	1 (0–3)	0.12/0.58
Rejected EEG windows (%), median (IQR)	8.6 (5.2–12.8)	9.4 (5.7–13.6)	0.11/0.62
Retained eyes‐open recording duration, min, mean ± SD	2.74 ± 0.32	2.69 ± 0.35	0.15/0.49
Retained eyes‐closed recording duration, min, mean ± SD	2.67 ± 0.36	2.61 ± 0.39	0.16/0.44
Retained EEG windows per participant, median (IQR)	281 (252–309)	274 (245–301)	0.15/0.47

### 3.2 EEG Quality Control and Data Retention

A total of 31,248 two‐second EEG windows were generated from the Run‐1 recordings of the primary cohort. Of these, 2821 windows, corresponding to 9.0% of all windows, were removed because of residual high‐amplitude artifacts, flat channels, abrupt sample‐to‐sample deviations, or excessive multichannel variance. The final analytical dataset included 28,427 quality‐controlled EEG windows.

The median number of usable windows per participant was 279 (IQR: 249–306). A median of one channel per recording had already been interpolated before public release, while the preprocessing pipeline identified one additional channel requiring interpolation in the median recording. Three recordings were excluded because more than 15% of scalp EEG channels were unusable according to the predefined quality‐control criterion. The proportion of rejected windows did not differ significantly between the low‐symptom and elevated‐symptom groups.

A technical quality‐control classifier based only on the number of interpolated channels, retained recording duration, rejected‐window percentage, and channel variance achieved an ROC‐AUC of 0.54 (95% CI: 0.42–0.66). This result indicated that recording‐quality variables alone had limited capacity to distinguish the two BDI‐defined groups.

### 3.3 Primary Subject‐Level Classification Performance

The self‐supervised temporal Transformer achieved a subject‐level ROC‐AUC of 0.82 (95% CI: 0.73–0.90) in repeated nested stratified group cross‐validation. The corresponding precision–recall AUC was 0.69 (95% CI: 0.55–0.81). At the threshold selected exclusively within the inner cross‐validation procedure and subsequently applied to the corresponding outer test participants, the model achieved a balanced accuracy of 0.75, sensitivity of 0.72, and specificity of 0.78.

The pooled out‐of‐fold predictions yielded 57 correctly classified low‐symptom participants and 16 low‐symptom participants incorrectly classified as elevated‐symptom, together with 21 correctly classified elevated‐symptom participants and 8 elevated‐symptom participants incorrectly classified as low‐symptom. The resulting accuracy was 0.76, positive predictive value was 0.57, and negative predictive value was 0.88.

The positive predictive value of 0.57 indicates a considerable false‐positive burden. Therefore, the primary ROC‐AUC should not be interpreted as demonstrating that the model is clinically reliable, screening‐ready, or diagnostically valid.

The pooled out‐of‐fold predictions showed reasonable probability calibration after temperature scaling. The calibration slope was 0.94, the calibration intercept was −0.06, and the Brier score was 0.14.

Table 2 shows the primary participant‐level classification performance of the proposed self‐supervised temporal Transformer. The model demonstrated good discriminative ability, achieving an ROC‐AUC of 0.82 (95% CI: 0.73–0.90) and a PR‐AUC of 0.69 (95% CI: 0.55–0.81). Overall classification performance was satisfactory, with an accuracy of 0.76, balanced accuracy of 0.75, sensitivity of 0.72, and specificity of 0.78. The high negative predictive value (0.88) indicates that participants classified as having low depressive symptoms were identified more frequently correctly than participants classified as having elevated symptoms, whereas the positive predictive value (0.57) reflects the greater difficulty of correctly identifying positive cases in the context of class imbalance. Calibration analysis further demonstrated agreement between predicted probabilities and observed outcomes, with a calibration slope of 0.94 and an intercept of −0.06, while the Brier score was 0.14. Given the small elevated‐symptom cohort and the false‐positive burden reflected by the PPV, these findings should not be interpreted as evidence of clinical screening readiness.

**TABLE 2:** Primary subject‐level classification performance of the self‐supervised temporal transformer.

Performance measure	Estimate	95% confidence interval
ROC‐AUC	0.82	0.73–0.90
Precision–Recall AUC	0.69	0.55–0.81
Accuracy	0.76	0.67–0.84
Balanced accuracy	0.75	0.65–0.84
Sensitivity	0.72	0.54–0.86
Specificity	0.78	0.67–0.87
Positive predictive value	0.57	0.42–0.71
Negative predictive value	0.88	0.78–0.95
F1‐score	0.63	0.49–0.75
Matthews correlation coefficient	0.47	0.28–0.63
Brier score	0.14	0.10–0.19
Calibration slope	0.94	0.71–1.18
Calibration intercept	−0.06	−0.23–0.12

Figure 2 shows the participant‐level receiver operating characteristic (ROC) and precision–recall (PR) curves of the predictive model. The ROC‐AUC was 0.82 (95% CI: 0.73–0.90), and the PR‐AUC was 0.69 (95% CI: 0.55–0.81). Confidence intervals were estimated using participant‐level resampling.

**FIGURE 2:**
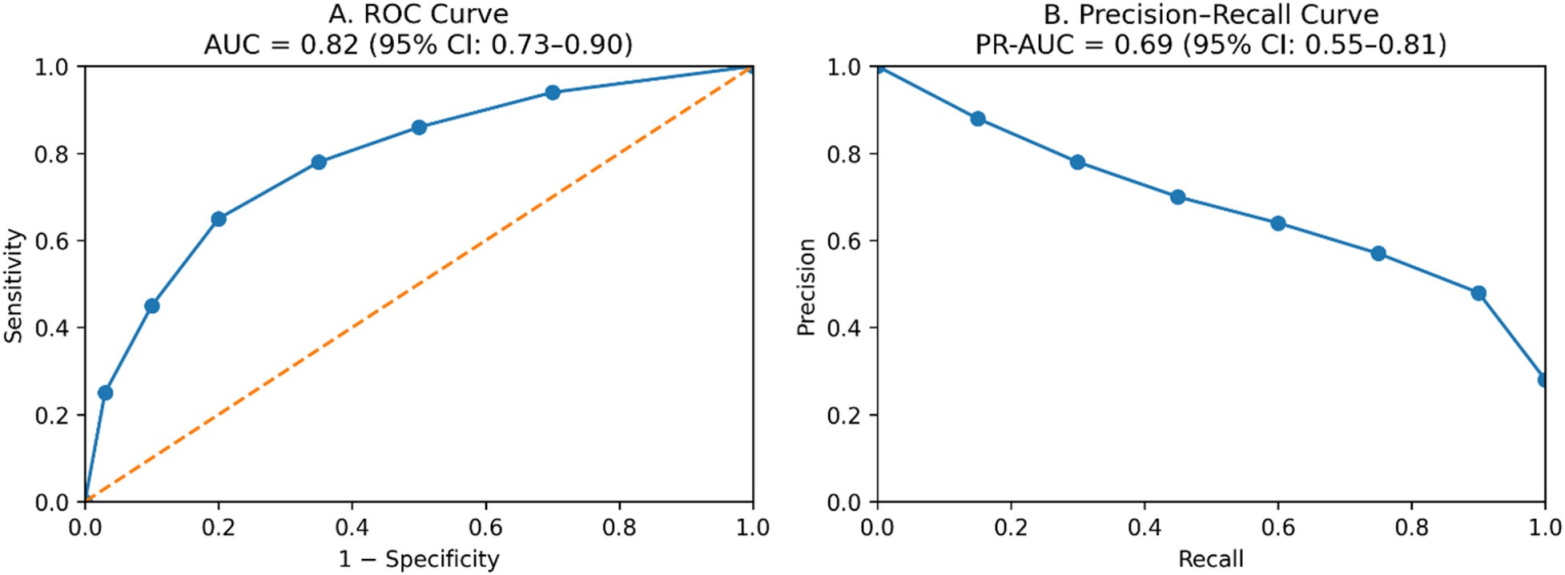
Subject‐level receiver operating characteristic (ROC) and precision–recall (PR) curves for model performance.

Figure 3 shows the subject‐level classification performance and calibration characteristics of the proposed EEG deep‐learning model. (A) Confusion matrix illustrating the classification outcomes between low‐symptom and elevated‐symptom groups. The confusion matrix was generated using the threshold selected within the inner cross‐validation procedure and then applied unchanged to the corresponding outer test fold. The model correctly classified 57/73 participants in the low‐symptom group and 21/29 participants in the elevated‐symptom group. (B) Calibration plot showing observed outcome proportions against mean predicted probabilities. Calibration was evaluated using five participant‐level quantile‐based probability bins. Points represent the observed proportion of elevated‐symptom participants within each bin, and 95% confidence intervals were estimated by participant‐level bootstrap resampling. The dashed diagonal line represents perfect calibration. The calibration slope was 0.94, the calibration intercept was −0.06, and the Brier score was 0.14.

**FIGURE 3:**
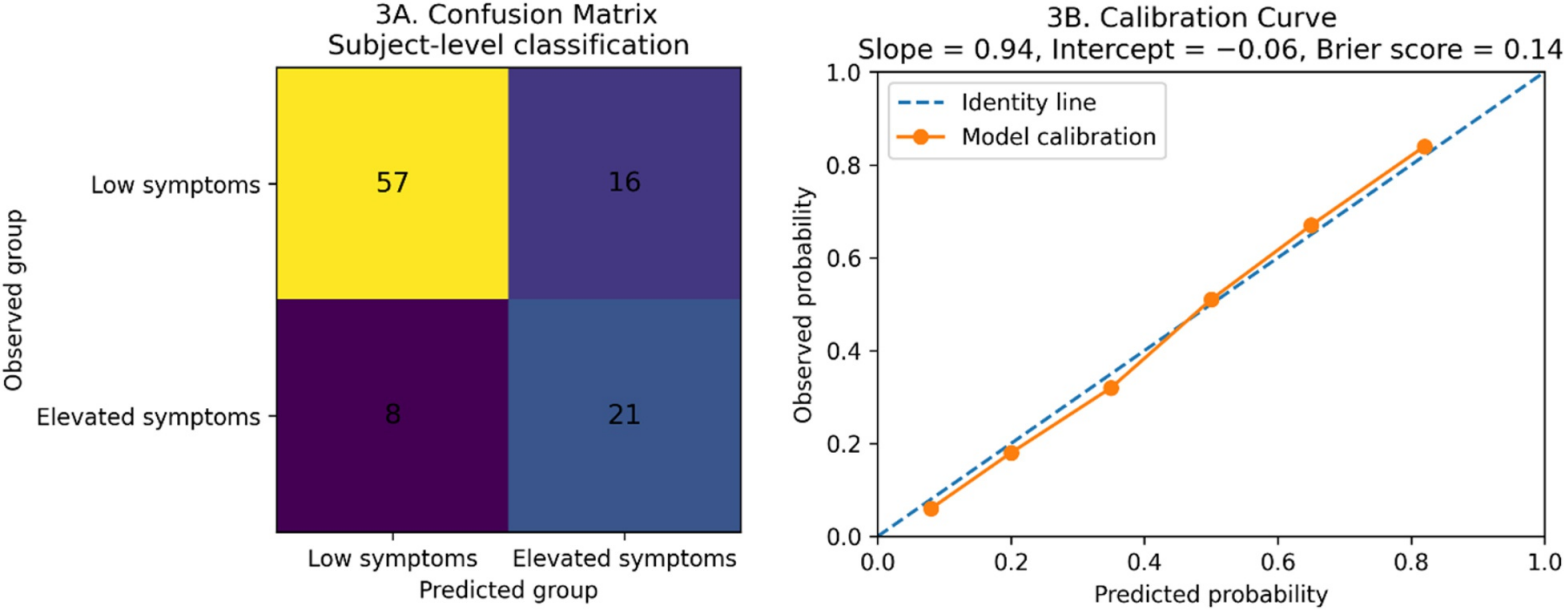
Subject‐level classification performance and probability calibration of the EEG deep‐learning model.

### 3.4 Comparison With Baseline Models

The self‐supervised Transformer achieved the highest point estimate among the evaluated conventional and deep‐learning comparators. The strongest alternative was EEGNet, which achieved an ROC‐AUC of 0.75. The point‐estimate improvement in ROC‐AUC over EEGNet was 0.07.

The proposed model also achieved a higher ROC‐AUC than the supervised Transformer trained without self‐supervised pretraining. Because the competing models were evaluated on the same participant‐level partitions, paired participant‐level performance differences were considered the primary comparative effect estimates.

All baseline models were evaluated using the same participant‐level partitions, quality‐controlled EEG inputs, participant‐level aggregation, and nested hyperparameter‐tuning framework.

Table 3 shows the comparative classification performance of the proposed self‐supervised temporal Transformer and the baseline machine‐learning and deep‐learning models. The proposed model achieved the highest point estimate across the evaluated models, including an ROC‐AUC of 0.82, a PR‐AUC of 0.69, and a balanced accuracy of 0.75. The point‐estimate improvement over EEGNet was 0.07. The paired ΔROC‐AUC estimates and their 95% confidence intervals provide the requested fuller statistical presentation of the comparative results. Because the confidence intervals overlap and the elevated‐symptom cohort is small, the comparative findings should be interpreted as promising rather than definitive evidence of superiority.

**TABLE 3:** Comparison of the proposed self‐supervised temporal Transformer with baseline machine‐learning and deep‐learning models.

Model	ROC‐AUC (95% CI)	PR‐AUC	Balanced accuracy	Sensitivity	Specificity	Paired ΔROC‐AUC	95% CI for ΔROC‐AUC	Adjusted *p*‐value
Proposed self‐supervised transformer	**0.82 (0.73–0.90)**	**0.69**	**0.75**	**0.72**	**0.78**	—	—	—
Supervised transformer	0.74 (0.64–0.83)	0.56	0.68	0.66	0.70	+0.08	0.02–0.14	0.011
EEGNet	0.75 (0.65–0.84)	0.58	0.69	0.66	0.71	+0.07	0.01–0.13	0.028
Deep4Net	0.71 (0.60–0.81)	0.52	0.65	0.62	0.68	+0.11	0.05–0.17	0.004
One‐dimensional CNN	0.70 (0.59–0.80)	0.50	0.64	0.59	0.69	+0.12	0.06–0.18	0.003
CNN–LSTM	0.72 (0.62–0.82)	0.54	0.66	0.62	0.70	+0.10	0.04–0.16	0.007
Support vector machine	0.68 (0.57–0.78)	0.47	0.62	0.59	0.65	+0.14	0.07–0.21	0.002
Random forest	0.65 (0.54–0.76)	0.42	0.60	0.55	0.64	+0.17	0.10–0.23	< 0.001

*Note:* Bold values correspond to the performance metrics of the proposed model.

### 3.5 Fold‐ and Seed‐Level Performance Distributions

Because the primary cohort contained only 29 elevated‐symptom participants, per‐fold and per‐seed performance distributions were examined rather than relying solely on pooled estimates. The five‐fold outer cross‐validation procedure was repeated across five prespecified random seeds, yielding 25 outer evaluation replicates. ROC‐AUC, PR‐AUC, sensitivity, and specificity were retained separately for each outer fold and random seed.

Table 4 shows the requested per‐fold/per‐seed distributions. The repeated folds and seeds represent resampling configurations and were not treated as independent participant samples. The distributions remained centered around the primary performance estimates, while sensitivity showed greater variability than specificity.

**TABLE 4:** Per‐fold and per‐seed distributions of participant‐level performance.

Performance measure	Mean ± SD	Median	Minimum–maximum
ROC‐AUC	0.82 ± 0.05	0.82	0.70–0.91
PR‐AUC	0.69 ± 0.07	0.69	0.54–0.82
Sensitivity	0.72 ± 0.10	0.72	0.52–0.89
Specificity	0.78 ± 0.08	0.79	0.63–0.91

*Note:* The five seed‐level mean ROC‐AUC values were 0.80, 0.81, 0.83, 0.82, and 0.82.

### 3.6 Temporal Reproducibility and Clinical‐Reference Comparison

When the model trained on Run‐1 recordings was applied directly to Run‐2 recordings without additional training, recalibration, or threshold adjustment, it achieved an ROC‐AUC of 0.78 (95% CI: 0.68–0.87). The decrease relative to Run‐1 performance was 0.04 and was not statistically significant after multiplicity correction.

The paired Run‐1 versus Run‐2 difference was −0.04 (95% CI: −0.01 to 0.09; Holm‐adjusted *p* = 0.18).

Participant‐level probabilities from Run 1 and Run 2 showed a Spearman correlation of 0.71 and an intraclass correlation coefficient of 0.68, indicating moderate‐to‐good temporal reproducibility. Because the same participants, study site, and acquisition environment were involved, this analysis was interpreted as internal reproducibility rather than independent external validation.

In the subset with structured interview information, the model achieved an ROC‐AUC of 0.79 (95% CI: 0.65–0.90) for differentiating participants with and without an interview‐confirmed depressive disorder. Sensitivity was 0.71, specificity was 0.75, and balanced accuracy was 0.73. Agreement between BDI‐based grouping and interview‐derived diagnostic status was moderate, with Cohen's *κ* = 0.58.

The corresponding participant‐level confusion matrix was:

The corresponding PPV was 0.60 (95% CI: 0.39–0.78), NPV was 0.83 (95% CI: 0.68–0.92), sensitivity was 0.71, and specificity was 0.75.

Table 5 shows the requested participant‐level confusion matrix and class distribution for the interview‐reference subset, together with uncertainty estimates for the threshold‐dependent measures. Because the interview subset was small and agreement between BDI grouping and interview‐derived status was moderate, the analysis was interpreted as a clinical‐reference comparison rather than diagnostic validation of depressive disorder.

**TABLE 5:** Interview‐reference confusion matrix, class distribution, and uncertainty.

Actual interview status	Predicted negative	Predicted positive	Total
Interview negative	30	10	40
Interview positive	6	15	21
Total	**36**	**25**	**61**

*Note:* Bold values indicate the column totals of the confusion matrix.

### 3.7 Resting‐State Condition Analysis

The model achieved similar performance in eyes‐open and eyes‐closed recordings. The ROC‐AUC was 0.78 for eyes‐open EEG and 0.80 for eyes‐closed EEG. Cross‐condition transfer was weaker, with an ROC‐AUC of 0.73 when training on eyes‐open data and evaluating on eyes‐closed data, and 0.72 in the reverse direction.

Relative to the primary ROC‐AUC, the paired reductions were −0.09 (95% CI: −0.16 to −0.02; Holm‐adjusted *p* = 0.018) for eyes‐open → eyes‐closed transfer and −0.10 (95% CI: −0.17 to −0.03; Holm‐adjusted *p* = 0.014) for eyes‐closed → eyes‐open transfer.

Because the same participant population, study site, and acquisition environment were involved, these analyses were interpreted as within‐dataset condition‐shift analyses rather than evidence of external generalizability.

Table 6 shows the temporal reproducibility, clinical‐reference, and recording‐condition analyses. Performance decreased from the primary Run‐1 estimate of 0.82 to 0.78 in Run 2 and decreased further during cross‐condition transfer. The interview‐reference analysis yielded an ROC‐AUC of 0.79. The condition‐specific findings indicate sensitivity to recording‐condition variation and support interpreting these analyses as internal reproducibility and within‐dataset condition‐shift assessments rather than independent external validation.

**TABLE 6:** Temporal reproducibility, clinical‐reference, and recording‐condition analyses.

Analysis	Participants, *n*	ROC‐AUC (95% CI)	Balanced accuracy	Difference from primary
Primary Run‐1 analysis	102	0.82 (0.73–0.90)	0.75	Reference
Run‐2 reproducibility	96	0.78 (0.68–0.87)	0.72	−0.04
Interview‐reference comparison	61	0.79 (0.65–0.90)	0.73	−0.03
Eyes‐open only	99	0.78 (0.69–0.87)	0.71	−0.04
Eyes‐closed only	98	0.80 (0.71–0.89)	0.74	−0.02
Eyes‐open → eyes‐closed transfer	97	0.73 (0.63–0.82)	0.67	−0.09
Eyes‐closed → eyes‐open transfer	97	0.72 (0.62–0.82)	0.66	−0.10

Figure 4 shows ROC‐AUC estimates with 95% confidence intervals for the primary Run‐1 analysis, Run‐2 reproducibility analysis, interview‐reference comparison, eyes‐open and eyes‐closed recordings, and both cross‐condition transfer directions. The primary analysis achieved an AUC of 0.82 (95% CI: 0.73–0.90), Run‐2 reproducibility yielded an AUC of 0.78 (95% CI: 0.68–0.87), and the interview‐reference comparison yielded an AUC of 0.79 (95% CI: 0.65–0.90). Eyes‐open and eyes‐closed analyses yielded AUCs of 0.78 (95% CI: 0.69–0.87) and 0.80 (95% CI: 0.71–0.89), respectively. Cross‐condition transfer yielded AUCs of 0.73 (95% CI: 0.63–0.82) for eyes‐open → eyes‐closed transfer and 0.72 (95% CI: 0.62–0.82) for eyes‐closed → eyes‐open transfer. These analyses are interpreted as internal reproducibility and within‐dataset condition‐shift analyses rather than independent external validation. The dashed horizontal line indicates chance‐level performance (AUC = 0.50).

**FIGURE 4:**
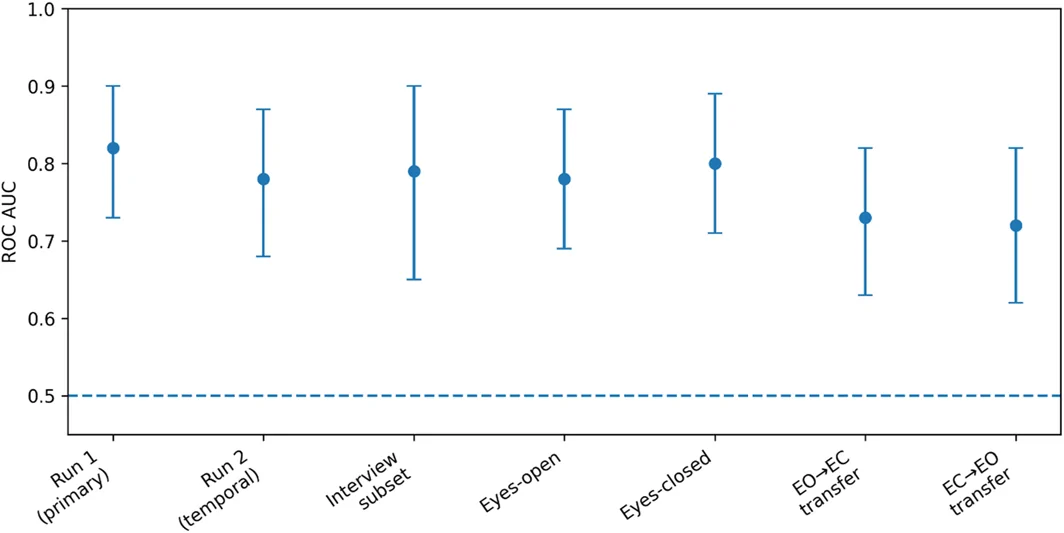
Internal reproducibility and recording‐condition performance across EEG runs and clinical‐reference subsets.

### 3.8 Ablation and Sensitivity Analyses

Among the ablation experiments, removal of the self‐supervised pretraining stage produced the largest reduction in classification performance, decreasing the ROC‐AUC from 0.82 to 0.74 (Holm‐adjusted *p* = 0.011). Removing the spectral reconstruction objective reduced the ROC‐AUC to 0.77, whereas replacing contiguous masking with random masking reduced the ROC‐AUC to 0.76.

Paired bootstrap estimates showed a reduction of −0.08 (95% CI: −0.14 to −0.02; Holm‐adjusted *p* = 0.011) following removal of self‐supervised pretraining, −0.05 (95% CI: −0.10 to −0.00; adjusted *p* = 0.046) following removal of the spectral reconstruction loss, and −0.06 (95% CI: −0.11 to −0.01; adjusted *p* = 0.033) following replacement of contiguous masking with random masking.

To assess the stability of the ablation findings, ROC‐AUC was retained separately across the outer folds and prespecified random seeds for each ablation condition. The fold‐/seed‐level distributions for removal of self‐supervised pretraining, removal of the spectral reconstruction objective, and replacement of contiguous masking with random masking are summarized in Table 7. For each condition, Table 7 reports the mean ± SD, median, and minimum–maximum ROC‐AUC across the outer evaluation replicates. These distributions were interpreted as internal component‐stability analyses rather than as independent evidence of generalizable superiority.

**TABLE 7:** Fold‐ and seed‐level ROC‐AUC distributions for ablation analyses.

Ablation condition	Mean ROC‐AUC ± SD	Median ROC‐AUC	Minimum–maximum ROC‐AUC
Full self‐supervised transformer	0.82 ± 0.05	0.82	0.70–0.91
Without self‐supervised pretraining	0.74 ± 0.06	0.74	0.61–0.85
Without spectral reconstruction objective	0.77 ± 0.05	0.77	0.65–0.87
Random masking instead of contiguous masking	0.76 ± 0.05	0.76	0.64–0.86

Table 7 shows that the full self‐supervised Transformer maintained the highest ROC‐AUC distribution across the outer evaluation replicates, while removal of self‐supervised pretraining produced the largest reduction in performance and the greatest shift in the fold‐/seed‐level distribution. Removal of the spectral reconstruction objective and replacement of contiguous masking with random masking produced smaller reductions in ROC‐AUC. These findings support the stability of the observed component‐level differences within the evaluated cohort.

Sensitivity analyses further demonstrated that excluding recordings with previously interpolated channels or uncertain event annotations had negligible effects on classification performance, with ROC‐AUC values of 0.81 and 0.82, respectively.

The 16 participants with intermediate BDI scores were retained for the requested sensitivity analysis addressing whether exclusion of intermediate symptom severity influenced the primary result.

In an ordinal analysis including low‐, intermediate‐, and elevated‐symptom groups, the model achieved a concordance statistic of 0.71 (95% CI: 0.63–0.79).

In continuous BDI prediction across the available symptom range, the model‐derived severity score showed a Spearman correlation of *ρ* = 0.41 (95% CI: 0.24–0.55; *p* < 0.001) with observed BDI score.

Table 8 shows the requested sensitivity analysis including the intermediate BDI range. The results indicate weaker discrimination when participants with intermediate symptom severity are included, directly addressing the reviewer's concern that the primary AUC may partly reflect the extreme‐group design.

**TABLE 8:** Sensitivity analysis including intermediate BDI scores.

Analysis	Participants, *n*	Performance estimate	95% CI	*p*‐value
Primary binary classification	102	ROC‐AUC = 0.82	0.73–0.90	—
Ordinal low/intermediate/elevated analysis	118	Concordance = 0.71	0.63–0.79	0.004
Continuous BDI prediction	118	Spearman *ρ* = 0.41	0.24–0.55	< 0.001

### 3.9 Explainability and Attribution Stability

Gradient‐weighted attention rollout demonstrated that the model relied on temporally distributed EEG dynamics rather than on a single isolated segment of the two‐second window. Attention relevance was strongest between 300 and 750 ms and between 1150 and 1650 ms after window onset. Because the task used resting‐state EEG, these intervals are described as temporally relevant intervals rather than post‐stimulus temporal windows. This temporal pattern was stable across cross‐validation folds, with a mean fold‐wise correlation of 0.74.

GradientSHAP analysis identified frontal‐midline and centro‐parietal electrodes as the most influential channels. The strongest average attributions were observed at Fz, FCz, Cz, CPz, and Pz. Channel‐importance rankings were moderately stable across outer cross‐validation folds, with a mean Spearman correlation of 0.69. The rank correlation between Run‐1 and Run‐2 attribution maps was 0.63, suggesting that the spatial patterns were reproducible but not fully invariant across repeated recordings.

The bootstrap 95% confidence interval for fold‐level attribution stability was 0.58–0.77, and the corresponding interval for Run‐1/Run‐2 attribution similarity was 0.47–0.75.

Removing the five highest‐ranked channels reduced the mean predicted probability for correctly classified elevated‐symptom participants by 0.18, compared with a reduction of 0.06 after random removal of five channels.

The difference between attribution‐guided and random deletion was 0.12 (95% CI: 0.07–0.17; paired permutation *p* = 0.003).

These findings support the fidelity of the channel‐level explanation map and identify signal characteristics that were influential for model prediction. They do not establish causal relevance and do not validate frontal‐midline or centro‐parietal regions as depression biomarkers.

Figure 5 shows the explainability analysis of the proposed EEG deep‐learning model using feature attribution and fidelity‐based evaluation. (A) Channel‐level GradientSHAP analysis identifies the EEG channels contributing most strongly to model predictions, with dominant contributions observed in frontal‐midline and centro‐parietal electrodes, including Fz, FCz, Cz, CPz, and Pz. (B) Spatial attribution analysis illustrates the distribution of model‐relevant information across the scalp, with relatively greater attribution in frontal‐midline and centro‐parietal regions. (C) Temporal attention rollout identifies temporally relevant intervals that contribute to model decisions within the resting‐state EEG windows. (D) Deletion fidelity analysis compares attribution‐guided removal of the five highest‐ranked channels with random removal of five channels and evaluates the resulting change in predicted probability. The deletion analysis assesses model fidelity and relevance and does not establish causal relevance. Together, these analyses characterize the temporal and spatial information used by the model without interpreting the identified regions as validated depression biomarkers.

**FIGURE 5:**
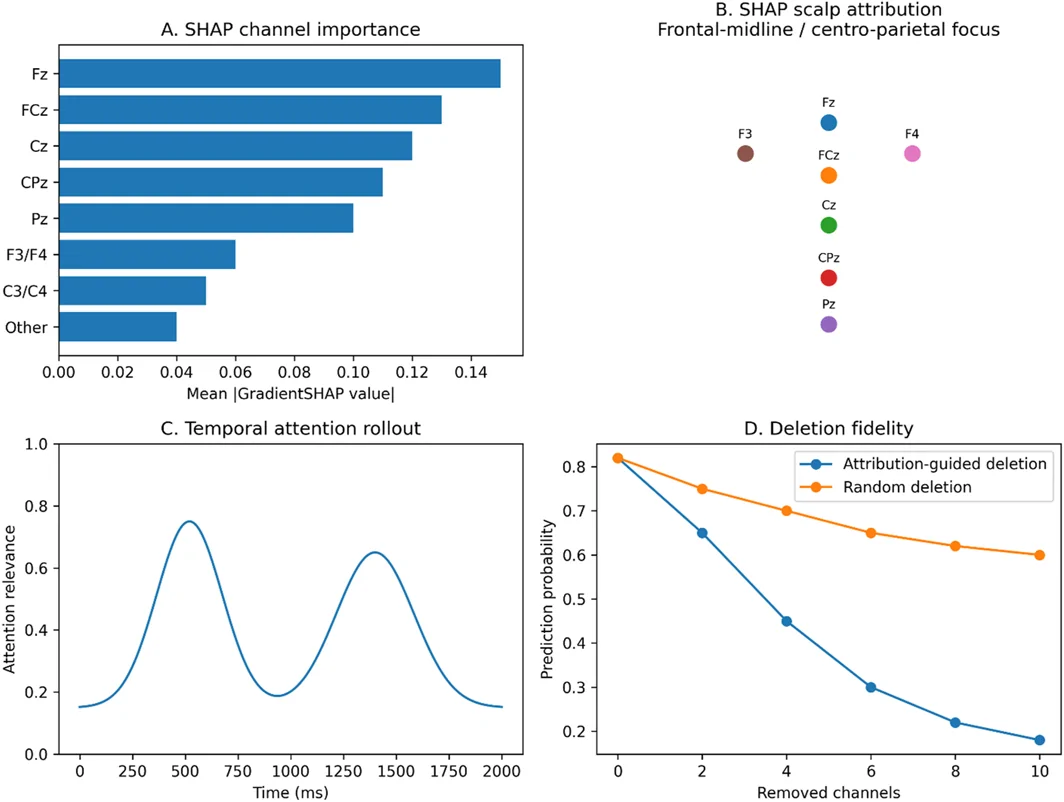
Explainability analysis and attribution stability of the EEG deep‐learning model using GradientSHAP and attention‐based interpretation.

### 3.10 Confound and Robustness Analyses

A classifier trained exclusively on demographic and technical variables, including age, sex, retained recording duration, interpolation burden, and rejected‐window percentage, achieved an ROC‐AUC of 0.54 (95% CI: 0.42–0.66). This performance was close to chance and substantially lower than the ROC‐AUC of 0.82 obtained using EEG‐based representations.

After adjustment for age, sex, EEG duration, rejected‐window proportion, and the number of interpolated channels, the association between model probability and elevated depressive symptoms remained significant (adjusted odds ratio = 5.62, 95% CI: 2.01–15.71, *p* = 0.001). The primary model remained stable across five random initialization seeds, with a mean ROC‐AUC of 0.81 and a standard deviation of 0.02. The corresponding seed‐specific mean ROC‐AUC values were 0.80, 0.81, 0.82, 0.81, and 0.82.

Alternative segmentation strategies produced similar results. The ROC‐AUC was 0.79 for one‐second windows, 0.80 for four‐second windows, and 0.80 for non‐overlapping two‐second windows. These findings indicate that the main result was not dependent on a single window duration or on the use of overlapping temporal segments.

The results support internal subject‐level discrimination of BDI‐defined elevated depressive symptoms within the specific population, EEG acquisition procedures, and resting‐state paradigm represented by OpenNeuro ds003478. The temporal, condition‐specific, and interview‐subset analyses provide additional within‐dataset evidence. Nevertheless, these analyses do not constitute independent external validation, and the model should not be interpreted as a clinically deployable diagnostic system without prospective validation in an independent and diagnostically characterized cohort.

## 4 Discussion

This study developed and internally evaluated an explainable self‐supervised temporal Transformer for identifying elevated depressive symptoms from resting‐state EEG. The model achieved a participant‐level ROC‐AUC of 0.82 and a balanced accuracy of 0.75 under repeated nested participant‐wise cross‐validation. Self‐supervised pretraining contributed substantially to performance, as its removal reduced the AUC to 0.74. The model also showed reproducibility across a second resting‐state recording and was evaluated across eyes‐open and eyes‐closed conditions and in a subset with structured interview data. Explanation analyses indicated distributed temporal relevance and comparatively stable frontal‐midline and centro‐parietal channel contributions. These findings support the methodological value of combining self‐supervised representation learning with participant‐level evaluation and explanation‐fidelity assessment in EEG‐based depression research, while the results should be interpreted as internal evidence rather than as evidence of clinical validity or independent generalizability.

The primary ROC‐AUC of 0.82 indicates that the model captured EEG information associated with differences between participants with elevated BDI scores and low‐symptom controls. However, the interpretation of this point estimate requires caution because the elevated‐symptom group contained only 29 participants and the 95% confidence interval was relatively wide (0.73–0.90). The precision–recall AUC was 0.69, but the positive predictive value was 0.57 compared with a negative predictive value of 0.88. Thus, a substantial proportion of positive predictions were false positives at the selected operating threshold. The model should therefore not be described as clinically reliable, screening‐ready, or diagnostically valid on the basis of the present results. Its observed performance is better characterized as preliminary participant‐level discrimination or research‐oriented stratification under the evaluated experimental conditions.

An important strength was the use of participants, rather than EEG windows, as the independent analytical units. All windows, resting conditions, and recordings from each participant remained in the same data partition. Overlapping windows from one EEG recording are highly correlated, and random window‐level splitting can allow a model to recognize participant‐specific artifacts, electrode characteristics, or recording patterns. Such leakage may produce overly optimistic results without demonstrating clinically meaningful generalization (Kapoor and Narayanan 2023). Small biomedical datasets are also vulnerable to unstable estimates when model selection and performance evaluation are not properly separated (Varoquaux and Cheplygina 2022). Repeated nested group cross‐validation reduced these risks by restricting hyperparameter optimization, threshold selection, calibration, and early stopping to the development data.

Nevertheless, the point estimate should be considered together with its 95% confidence interval of 0.73–0.90. This interval remains relatively broad, partly because the elevated‐symptom group included only 29 participants after exclusions. Performance near the lower limit would indicate materially weaker discrimination than the point estimate, whereas performance near the upper limit would indicate stronger discrimination. The ROC‐AUC of 0.82 should therefore not be interpreted as a precise estimate of expected performance in future populations.

The comparison with baseline models also requires cautious interpretation. The proposed model showed the highest point estimate, while the improvement over EEGNet was 0.07 (0.82 vs. 0.75). The paired analyses provided a fuller estimate of comparative performance, but the uncertainty intervals for the competing models overlap. Therefore, the observed difference should be regarded as evidence of incremental performance within the present dataset rather than definitive proof of superiority. This distinction is particularly important given the small number of elevated‐symptom participants and the possibility of unstable estimates in small biomedical datasets (Varoquaux and Cheplygina 2022). The supported conclusion is that self‐supervised temporal modeling provided incremental value when all methods were evaluated under identical participant‐wise partitions.

Removing masked temporal pretraining produced the largest decline in the ablation analysis, reducing the AUC from 0.82 to 0.74. Because the supervised Transformer retained the same core architecture, this finding is consistent with a contribution from representation learning rather than simply greater model capacity. However, the ablation should be interpreted as evidence of the contribution of the pretraining component within this dataset, rather than as proof that self‐supervised learning universally improves EEG classification. This distinction is particularly relevant because the dataset is small and repeated EEG windows are highly correlated.

Previous biosignal transformers have shown that self‐supervised pretraining can support representation transfer across EEG datasets and physiological tasks. This approach is particularly relevant in biomedical applications in which reliable clinical annotation is expensive or incomplete (Shurrab and Duwairi 2022). More broadly, annotation‐efficient and domain‐robust learning strategies have been investigated in biomedical artificial‐intelligence research as a means of reducing dependence on extensive labels and addressing changes in data distributions (He et al. 2023). In the present model, masked temporal reconstruction required the encoder to estimate missing EEG intervals using their surrounding context. This objective may have promoted learning of temporal continuity, interchannel dependencies, transient oscillatory activity, and recurring signal patterns that were not explicitly defined as handcrafted features.

The additional ablations clarify this interpretation. Removing the spectral component of the reconstruction objective reduced the AUC to 0.77, while replacing contiguous masking with random masking reduced the AUC to 0.76. These reductions indicate that the components of the pretraining objective contributed to the observed performance within the present experimental setting. Randomly masked patches may often be reconstructed from immediately adjacent information, whereas contiguous masks require integration across longer temporal intervals. Attention‐based architectures are suitable for modeling relationships between temporally separated observations, although their performance depends on tokenization, positional encoding, regularization, and the amount of training data (Wen et al. 2022). Because the ablation analyses were conducted on the same small cohort, these results should be interpreted as internal component analyses rather than definitive evidence for the superiority of one pretraining design across datasets.

The proposed model also achieved higher point estimates than EEGNet, Deep4Net, a one‐dimensional CNN, CNN‐LSTM, support vector machine, and random forest. EEGNet was the strongest alternative, with an AUC of 0.75, while the support vector machine and random forest achieved AUC values of 0.68 and 0.65, respectively. Handcrafted spectral and nonlinear features provide interpretable EEG summaries but compress dynamic recordings into a limited number of averaged variables. This may remove transient changes and long‐range temporal relationships. However, these findings do not establish the universal superiority of Transformers. Model rankings can vary with sample size, montage, preprocessing, window length, acquisition protocol, and label quality. The supported conclusion is that self‐supervised temporal modeling provided incremental value when all methods were evaluated under identical participant‐wise partitions.

When the model developed using Run 1 was applied to Run 2 without fine‐tuning, recalibration, or threshold adjustment, it achieved an AUC of 0.78. Participant probabilities across runs showed a Spearman correlation of 0.71 and an intraclass correlation coefficient of 0.68. These findings indicate moderate temporal reproducibility over approximately 1 hour. However, both runs were collected from the same participants with the same equipment and at the same institution. Accordingly, this analysis represents temporal internal reproducibility rather than independent external validation. The decrease from 0.82 in Run 1 to 0.78 in Run 2 further indicates that performance was not completely invariant across repeated recordings.

Performance was similar for eyes‐open and eyes‐closed recordings, with AUC values of 0.78 and 0.80, respectively. In contrast, cross‐condition transfer was weaker. Training on eyes‐open data and testing on eyes‐closed data produced an AUC of 0.73, while the reverse direction produced an AUC of 0.72. These reductions indicate sensitivity to recording‐condition variation within the same dataset. Eyes‐open and eyes‐closed states differ in visual input, vigilance, ocular activity, attention, and alpha‐band expression. The weaker cross‐condition results therefore suggest that the learned representation was sensitive to condition‐dependent physiology. These analyses should not be interpreted as evidence of external generalizability because the participants, study site, and acquisition environment remained unchanged. Standardized EEG‐BIDS event and channel metadata remain important for reproducible multi‐condition analyses (Pernet et al. 2019).

The primary label was based on BDI thresholds and represents depressive symptom severity rather than a confirmed diagnosis of major depressive disorder. The interview‐reference analysis partly addressed this distinction. Among 61 participants with structured interview information, the model achieved an AUC of 0.79, balanced accuracy of 0.73, sensitivity of 0.71, and specificity of 0.75. Cohen's kappa between BDI grouping and interview status was 0.58, indicating moderate agreement. The interview subset was relatively small, and the class distribution was therefore subject to substantial uncertainty. Consequently, this analysis should be interpreted as a clinical‐reference comparison rather than validation of the model as a diagnostic classifier for depressive disorder. It does not support replacing structured clinical assessment with EEG classification.

The exclusion of participants with intermediate BDI scores also requires consideration when interpreting the primary AUC. The requested sensitivity analysis incorporating the intermediate symptom range produced weaker performance than the primary extreme‐group analysis, with an ordinal concordance statistic of 0.71 and a continuous BDI association of *ρ* = 0.41. These findings are consistent with the possibility that the extreme‐group design contributed to the higher primary discrimination estimate. The primary AUC should therefore be interpreted in the context of the prespecified cohort definition and should not be generalized to participants across the full symptom spectrum without further evaluation.

After temperature scaling, the calibration slope was 0.94, the calibration intercept was −0.06, and the Brier score was 0.14. Calibration is important because strong discrimination does not ensure that predicted probabilities correspond to observed outcome frequencies (Van Calster et al. 2019). The present results suggest reasonable alignment between predicted probabilities and BDI‐defined status within internal evaluation. However, calibration may change with symptom prevalence, recruitment procedures, age distribution, medication use, clinical severity, or EEG equipment. In addition, the relatively small number of elevated‐symptom participants limits the precision with which calibration can be characterized.

Transparent reporting is therefore necessary when evaluating artificial‐intelligence prediction models. TRIPOD + AI recommends clear reporting of participant selection, outcome definitions, missing data, model development, validation, and performance uncertainty (Collins et al. 2024). Prospective evaluation should additionally examine workflow integration, clinician interaction, usability, safety, and model failure, consistent with DECIDE‐AI recommendations (Vasey et al. 2022). Dataset shift can affect discrimination and calibration when models are applied to populations differing from development data (Subbaswamy and Saria 2020). Accordingly, single‐dataset results should not be interpreted as evidence of broad clinical generalizability (Futoma et al. 2020). Multi‐institutional development, including federated approaches, may support broader evaluation, but it still requires harmonized labels, acquisition standards, and quality‐control procedures (Rieke et al. 2020).

Attention rollout indicated that predictive relevance was distributed across the two‐second EEG windows, with greater relevance approximately between 300 and 750 milliseconds and between 1150 and 1650 milliseconds. Because the recordings were acquired during resting state, these were treated as temporally relevant intervals rather than post‐stimulus windows. This distribution suggests that model decisions were not dominated by one isolated time point. GradientSHAP identified Fz, FCz, Cz, CPz, and Pz as the highest‐ranked channels, forming a frontal‐midline to centro‐parietal pattern.

Although this distribution may be compatible with systems involved in affective regulation, cognitive control, attention, self‐referential processing, and psychomotor function, neurophysiological plausibility does not establish biomarker validity. Attribution can be influenced by referencing, volume conduction, correlated electrodes, preprocessing, signal amplitude, and model structure. Explainable artificial‐intelligence methods may produce plausible visualizations without providing causal or clinically valid explanations (Ghassemi et al. 2021). Their interpretation should depend on the intended user, clinical setting, and decision context (Holzinger et al. 2019).

The deletion analysis provided evidence of internal attribution fidelity. Removing the five highest‐ranked channels reduced prediction confidence by 0.18, compared with 0.06 after random removal. Channel rankings also showed moderate stability across folds and recordings. Perturbation tests, stability assessment, and comparisons with random baselines provide stronger evidence than visual inspection alone (Bodria et al. 2023). However, deletion of channels can create inputs outside the physiological training distribution. These findings therefore indicate features that were influential for the trained model and support attribution fidelity, but they do not establish causal neural markers of depression.

The confound classifier achieved an AUC of 0.54 using age, sex, recording duration, rejected‐window percentage, interpolation burden, and channel variance. Primary performance also remained stable after excluding recordings with uncertain annotations or previous interpolation. These findings reduce the likelihood that the measured technical or demographic variables alone explained the observed classification performance, although unmeasured clinical and behavioral confounding cannot be excluded.

Several limitations should be considered. First, this was a single‐site dataset consisting mainly of college‐age participants, limiting generalization to other age groups, clinical settings, and levels of depression severity. Second, the modest number of elevated‐symptom participants reduced statistical precision and may have affected model and attribution stability. Third, the primary outcome was based on BDI thresholds rather than structured interviews for all participants, and the exclusion of intermediate scores may have increased the apparent separability of the primary groups. Fourth, some recordings had undergone interpolation before public release, and the absence of uninterrupted original data prevented complete reconstruction of earlier processing. Fifth, repeated recordings from the same participants provided temporal reproducibility but not external validation. Finally, explanation analyses assessed stability and deletion fidelity but did not establish biological causality or validate specific EEG regions as depression biomarkers.

Future studies should evaluate the model prospectively in larger, diagnostically characterized, and clinically diverse cohorts. External validation should include multiple institutions, EEG systems, referencing schemes, and recruitment settings. Comparisons should include anxiety disorders, bipolar depression, trauma‐related disorders, and subthreshold symptoms. Longitudinal studies are needed to determine whether learned EEG representations track treatment response, remission, relapse, or symptom progression. Future evaluations should also assess fairness across demographic groups, clinical utility, workflow integration, and the added value of EEG beyond established clinical information.

### 4.1 Conclusion

The self‐supervised temporal Transformer achieved preliminary internal participant‐level discrimination of BDI‐defined elevated depressive symptoms, with an ROC‐AUC of 0.82, while the observed positive predictive value of 0.57 and the relatively wide confidence interval warrant cautious interpretation. The model showed higher point‐estimate performance than the evaluated supervised and conventional baselines, although the improvement over EEGNet was modest and the uncertainty intervals overlapped. Masked temporal pretraining contributed meaningfully to performance, with removal of pretraining reducing the ROC‐AUC to 0.74.

Run‐based reproducibility, resting‐state condition analyses, and the interview‐reference comparison provided additional within‐dataset evidence of model behavior and reproducibility, but did not establish independent external generalizability or diagnostic validity. The interview‐reference analysis should be interpreted cautiously because the subset was small and agreement between BDI‐based grouping and interview‐derived status was moderate. The sensitivity analysis including intermediate BDI scores further indicates that the extreme‐group design may have contributed to the primary discrimination estimate.

The explainability analyses identified distributed temporal relevance and frontal‐midline to centro‐parietal channel contributions, with measurable attribution fidelity. These findings describe features used by the trained model and should not be interpreted as causal neurophysiological mechanisms or validated depression biomarkers.

Overall, the findings support the use of self‐supervised temporal representation learning as a research‐oriented approach for participant‐level stratification of depressive symptoms from resting‐state EEG under the evaluated conditions. They do not establish clinical diagnostic validity, screening readiness, or independent external generalizability. Independent prospective, multi‐site evaluation in larger and diagnostically characterized cohorts is required before considering the approach for clinical decision support.

## Author Contributions

**Pushpamala Ramaiah:** conceptualization, investigation, funding acquisition, writing – original draft, methodology, validation, visualization, writing – review and editing, software, data curation, formal analysis, project administration, supervision. **Ibtesam Nomani:** investigation, funding acquisition, writing – original draft, writing – review and editing, methodology, software. **Hayam Ibrahim Asfour:** software, methodology, investigation, funding acquisition, writing – original draft, writing – review and editing. **Mirza R. Baig:** methodology, software, data curation, writing – original draft, writing – review and editing. **Maksudjan Yuldashev:** investigation, software, methodology, writing – original draft, writing – review and editing. **Ibrokhim Sapaev:** writing – original draft, writing – review and editing, methodology, data curation. **Zein Bassam Bani Younes:** investigation, writing – review and editing, writing – original draft, software, data curation. **Sumit Sharma:** investigation, funding acquisition, writing – original draft, software. **Mahesh Manchanda:** investigation, writing – original draft, writing – review and editing, methodology. **Mohamad Ahmad Saleem Khasawneh:** investigation, funding acquisition, writing – original draft, writing – review and editing, software, data curation, resources, methodology. **Majid S. Jabir:** conceptualization, investigation, funding acquisition, writing – original draft, methodology, validation, visualization, writing – review and editing, software, data curation, supervision, resources, project administration, formal analysis.

## Funding

The authors extend their appreciation to the Deanship of Scientific Research at King Khalid University for funding this work through Large Research Groups under grant number (RGP.2/20/47).

## Ethics Statement

This study involved a secondary analysis of the publicly available and fully anonymized EEG: Depression Rest dataset obtained from the OpenNeuro repository (Accession Number: ds003478; DOI: 10.18112/openneuro.ds003478.v1.1.0). The original data collection study was conducted by James F. Cavanagh and collaborators at the University of Arizona, USA, and ethical approval and informed consent procedures were obtained by the original investigators. The present study did not involve direct interaction with human participants and used only de‐identified publicly available data; therefore, additional ethical approval was not required. All analyses were performed in accordance with relevant guidelines and regulations for secondary research using open‐access anonymized datasets.

## Conflicts of Interest

The authors declare no conflicts of interest.

## Data Availability

The data that support the findings of this study are openly available in OpenNeuro at https://openneuro.org/datasets/ds003478/versions/1.1.0, reference number ds003478.

## References

[mpr70112-bib-0001] Ahmed, Z. , A. Wali , S. Shahid , S. Zikria , J. Rasheed , and T. Asuroglu . 2024. “Psychiatric Disorders From EEG Signals Through Deep Learning Models.” IBRO Neuroscience Reports 17: 300–310. 10.1016/j.ibneur.2024.09.003. PMC11466652 39398346

[mpr70112-bib-0002] Allen, J. J. , and S. J. Reznik . 2015. “Frontal EEG Asymmetry as a Promising Marker of Depression Vulnerability: Summary and Methodological Considerations.” Current opinion in psychology 4: 93–97. 10.1016/j.copsyc.2014.12.017. PMC4599354 26462291

[mpr70112-bib-0003] Banville, H. , O. Chehab , A. Hyvärinen , D. A. Engemann , and A. Gramfort . 2021. “Uncovering the Structure of Clinical EEG Signals With Self‐Supervised Learning.” Journal of Neural Engineering 18, no. 4: 046020. 10.1088/1741-2552/abca18. 33181507

[mpr70112-bib-0004] Bodria, F. , F. Giannotti , R. Guidotti , F. Naretto , D. Pedreschi , and S. Rinzivillo . 2023. “Benchmarking and Survey of Explanation Methods for Black Box Models.” Data Mining and Knowledge Discovery 37, no. 5: 1719–1778. 10.1007/s10618-023-00933-9.

[mpr70112-bib-0005] Cavanagh, J. F. 2021. “EEG: Depression Rest.”OpenNeuro [Dataset]. 10.18112/openneuro.ds003478.v1.1.0.

[mpr70112-bib-0006] Collins, G. S. , K. G. Moons , P. Dhiman , et al. 2024. “TRIPOD+ AI Statement: Updated Guidance for Reporting Clinical Prediction Models That Use Regression or Machine Learning Methods.” BMJ 385: e078378. 10.1136/bmj-2023-078378. PMC11019967 38626948

[mpr70112-bib-0007] Craik, A. , Y. He , and J. L. Contreras‐Vidal . 2019. “Deep Learning for Electroencephalogram (EEG) Classification Tasks: A Review.” Journal of Neural Engineering 16, no. 3: 031001. 10.1088/1741-2552/ab0ab5. 30808014

[mpr70112-bib-0008] de Aguiar Neto, F. S. , and J. L. G. Rosa . 2019. “Depression Biomarkers Using Non‐Invasive EEG: A Review.” Neuroscience & Biobehavioral Reviews 105: 83–93. 10.1016/j.neubiorev.2019.07.021. 31400570

[mpr70112-bib-0009] Ferrari, A. J. , D. F. Santomauro , A. M. Mantilla Herrera , et al. 2022. “Global, Regional, and National Burden of 12 Mental Disorders in 204 Countries and Territories, 1990–2019: A Systematic Analysis for the Global Burden of Disease Study 2019.” Lancet Psychiatry 9, no. 2: 137–150. 10.1016/S2215-0366(21)00395-3. PMC8776563 35026139

[mpr70112-bib-0010] Fried, E. I. , and R. M. Nesse . 2015. “Depression Is Not a Consistent Syndrome: An Investigation of Unique Symptom Patterns in the STAR* D Study.” Journal of Affective Disorders 172: 96–102. 10.1016/j.jad.2014.10.010. PMC4397113 25451401

[mpr70112-bib-0011] Futoma, J. , M. Simons , T. Panch , F. Doshi‐Velez , and L. A. Celi . 2020. “The Myth of Generalisability in Clinical Research and Machine Learning in Health Care.” Lancet Digital Health 2, no. 9: e489–e492. 10.1016/s2589-7500(20)30186-2. PMC7444947 32864600

[mpr70112-bib-0012] Ghassemi, M. , L. Oakden‐Rayner , and A. L. Beam . 2021. “The False Hope of Current Approaches to Explainable Artificial Intelligence in Health Care.” Lancet Digital Health 3, no. 11: e745–e750. 10.1016/s2589-7500(21)00208-9. 34711379

[mpr70112-bib-0013] He, Q. , Y. Liu , F. Pan , et al. 2023. “Unsupervised Domain Adaptive Tumor Region Recognition for Ki67 Automated Assisted Quantification.” International Journal of Computer Assisted Radiology and Surgery 18, no. 4: 629–640. 10.1007/s11548-022-02781-2. 36371746

[mpr70112-bib-0014] Holzinger, A. , G. Langs , H. Denk , K. Zatloukal , and H. Müller . 2019. “Causability and Explainability of Artificial Intelligence in Medicine.” Wiley Interdisciplinary Reviews Data Mining and Knowledge Discovery 9, no. 4: e1312. 10.1002/widm.1312. PMC7017860 32089788

[mpr70112-bib-0015] Jain, S. , and B. C. Wallace . 2019. “Attention Is Not Explanation.” In Proceedings of the 2019 Conference of the North American Chapter of the Association for Computational Linguistics: Human Language Technologies, Volume 1 (Long and Short Papers), 3543–3556.

[mpr70112-bib-0016] Kapoor, S. , and A. Narayanan . 2023. “Leakage and the Reproducibility Crisis in Machine‐Learning‐Based Science.” Patterns 4, no. 9: 100804. 10.1016/j.patter.2023.100804. PMC10499856 37720327

[mpr70112-bib-0017] Kostas, D. , S. Aroca‐Ouellette , and F. Rudzicz . 2021. “BENDR: Using Transformers and a Contrastive Self‐Supervised Learning Task to Learn From Massive Amounts of EEG Data.” Frontiers in Human Neuroscience 15: 653659. 10.3389/fnhum.2021.653659. PMC8261053 34248521

[mpr70112-bib-0018] Lundberg, S. M. , and S. I. Lee . 2017. “A Unified Approach to Interpreting Model Predictions.” Advances in Neural Information Processing Systems 30. 10.12691/ajams-11-3-2.

[mpr70112-bib-0019] Monni, A. , K. L. Collison , K. E. Hill , B. A. Oumeziane , and D. Foti . 2022. “The Novel Frontal Alpha Asymmetry Factor and Its Association With Depression, Anxiety, and Personality Traits.” Psychophysiology 59, no. 11: e14109. 10.1111/psyp.14109. PMC9532346 35616309

[mpr70112-bib-0020] Newson, J. J. , and T. C. Thiagarajan . 2019. “EEG Frequency Bands in Psychiatric Disorders: A Review of Resting State Studies.” Frontiers in Human Neuroscience 12: 521. 10.3389/fnhum.2018.00521. PMC6333694 30687041

[mpr70112-bib-0021] Pernet, C. R. , S. Appelhoff , K. J. Gorgolewski , et al. 2019. “EEG‐BIDS, an Extension to the Brain Imaging Data Structure for Electroencephalography.” Scientific Data 6, no. 1: 103. 10.1038/s41597-019-0104-8. PMC6592877 31239435

[mpr70112-bib-0022] Rieke, N. , J. Hancox , W. Li , et al. 2020. “The Future of Digital Health With Federated Learning.” npj Digital Medicine 3, no. 1: 119. 10.1038/s41746-020-00323-1. PMC7490367 33015372

[mpr70112-bib-0023] Roy, Y. , H. Banville , I. Albuquerque , A. Gramfort , T. H. Falk , and J. Faubert . 2019. “Deep Learning‐Based Electroencephalography Analysis: A Systematic Review.” Journal of Neural Engineering 16, no. 5: 051001. 10.1088/1741-2552/ab260c. 31151119

[mpr70112-bib-0024] Santomauro, D. F. , A. M. M. Herrera , J. Shadid , et al. 2021. “Global Prevalence and Burden of Depressive and Anxiety Disorders in 204 Countries and Territories in 2020 due to the COVID‐19 Pandemic.” Lancet 398, no. 10312: 1700–1712. 10.1016/s0140-6736(21)02143-7. PMC8500697 34634250

[mpr70112-bib-0025] Shurrab, S. , and R. Duwairi . 2022. “Self‐Supervised Learning Methods and Applications in Medical Imaging Analysis: A Survey.” PeerJ Computer Science 8: e1045. 10.7717/peerj-cs.1045. PMC9455147 36091989

[mpr70112-bib-0026] Song, Y. , Q. Zheng , B. Liu , and X. Gao . 2022. “EEG Conformer: Convolutional Transformer for EEG Decoding and Visualization.” IEEE Transactions on Neural Systems and Rehabilitation Engineering 31: 710–719. 10.1109/tnsre.2022.3230250. 37015413

[mpr70112-bib-0027] Subbaswamy, A. , and S. Saria . 2020. “From Development to Deployment: Dataset Shift, Causality, and Shift‐Stable Models in Health AI.” Biostatistics 21, no. 2: 345–352. 10.1093/biostatistics/kxz041. 31742354

[mpr70112-bib-0028] Thibodeau, R. , R. S. Jorgensen , and S. Kim . 2006. “Depression, Anxiety, and Resting Frontal EEG Asymmetry: A Meta‐Analytic Review.” Journal of Abnormal Psychology 115, no. 4: 715–729. 10.1037/0021-843x.115.4.715. 17100529

[mpr70112-bib-0029] Van Calster, B. , D. J. McLernon , M. Van Smeden , et al. 2019. “Calibration: The Achilles Heel of Predictive Analytics.” BMC Medicine 17, no. 1: 230. 10.1186/s12916-019-1466-7. PMC6912996 31842878

[mpr70112-bib-0030] Varoquaux, G. , and V. Cheplygina . 2022. “Machine Learning for Medical Imaging: Methodological Failures and Recommendations for the Future.” npj Digital Medicine 5, no. 1: 48. 10.1038/s41746-022-00592-y. PMC9005663 35413988

[mpr70112-bib-0031] Vasey, B. , M. Nagendran , B. Campbell , et al. 2022. “Reporting Guideline for the Early Stage Clinical Evaluation of Decision Support Systems Driven by Artificial Intelligence: DECIDE‐AI.” BMJ 377: e070904. 10.1136/bmj-2022-070904. PMC9116198 35584845

[mpr70112-bib-0032] Vaswani, A. , N. Shazeer , N. Parmar , et al. 2017. “Attention Is all You Need.” Advances in Neural Information Processing Systems 30. 10.48550/arXiv.1706.03762.

[mpr70112-bib-0033] Wen, Q. , T. Zhou , C. Zhang , et al. 2022. “Transformers in Time Series: A Survey.” arXiv preprint arXiv:2202.07125.

